# Targeting DESI2 as a Novel Therapeutic Strategy for JAK2‐Mutant Leukemias

**DOI:** 10.1002/advs.202515127

**Published:** 2025-12-03

**Authors:** Husheng Mei, Wuqiang Wen, Wenjun Zhang, Shujing Zhang, Ting Sun, Guiming Li, Jing Zhang, Shuang Qi, Jie Zhou, Bing Li, Yunshuo Zhao, Xiaotong Chen, Bowen Li, Yiying Xue, Wang Lu, Yanli Sun, Jingyao Wang, Hengyue Shan, Shengzhe Zhang, Yushan Huang, Yisa Chen, Wenchao Wang, Qingsong Liu, Wenchao Lu, Li Tan, Yi Ding, Jianfei Fu, Jun Long, Lei Zhang, Baobing Zhao, Aibin Liang, Baishan Jiang, Jing Yang

**Affiliations:** ^1^ Department of Hematology Tongji Hospital Frontier Science Center for Stem Cell Research Shanghai Key Laboratory of Signaling and Disease Research School of Life Sciences and Technology Tongji University Shanghai 200092 P. R. China; ^2^ Department of Radiation and Medical Oncology Medical Research Institute Frontier Science Center of Immunology and Metabolism Hubei Key Laboratory of Tumor Biological Behavior Zhongnan Hospital of Wuhan University Wuhan University Wuhan 430071 P. R. China; ^3^ Key Lab of Chemical Biology School of Pharmaceutical Sciences Cheeloo College of Medicine Shandong University Jinan Shandong 250012 P. R. China; ^4^ State Key Laboratory of Experimental Hematology National Clinical Research Center for Blood Diseases Haihe Laboratory of Cell Ecosystem Tianjin & CAMS Key Laboratory of Gene Therapy for Blood Diseases Institute of Hematology & Blood Diseases Hospital Chinese Academy of Medical Sciences & Peking Union Medical College Tianjin 300020 P. R. China; ^5^ Anhui Province Key Laboratory of Medical Physics and Technology Institute of Health and Medical Technology Hefei Institutes of Physical Science Chinese Academy of Sciences Hefei 230031 P. R. China; ^6^ Lingang Laboratory Shanghai 200031 P. R. China; ^7^ Interdisciplinary Research Center on Biology and Chemistry Shanghai Institute of Organic Chemistry Chinese Academy of Sciences Shanghai 201210 P. R. China; ^8^ Institute of Translational Medicine Medical College Yangzhou University Yangzhou 225001 P. R. China

**Keywords:** degrader, DESI2, drug resistance, JAK2‐V617F mutation, MPN

## Abstract

The JAK2‐V617F mutation is the most common genetic alteration in myeloproliferative neoplasms (MPN), which can progress to secondary acute myeloid leukemia (sAML), a chemotherapy‐resistant disease with limited treatment options and a poor prognosis. Although the JAK1/2 inhibitor Ruxolitinib is clinically approved, its efficacy is limited by toxicity to normal cells and the development of drug resistance. Here, the deSUMOylase DESI2 is identified as a novel component of the JAK2‐V617F complex by mass spectrometry‐based proteomics. Mechanistically, DESI2 selectively binds to and stabilizes JAK2‐V617F by mediating its deSUMOylation and deubiquitination at lysine 962 (K962). Importantly, DESI2 protein is specifically and highly expressed in JAK2‐mutant‐driven cell lines and MPN primary clinical samples, suggesting its potential role in JAK2‐V617F regulation and disease progression. Genetic depletion of DESI2 suppresses both JAK2 mutant cell growth and MPN disease onset in vitro and in vivo. Moreover, through a compound screen, followed by chemical proteomics and compound optimization, WWQ‐03‐012 is discovered, which selectively degrades mutant JAK2, induces primary leukemia cells death, and inhibits MPN progression through targeting DESI2 enzymatic activity in vitro and in vivo. These studies provide a novel therapeutic strategy against mutated JAK2 signaling in MPN and sAML.

## Graphical abstract

Mass spectrometry‐based proteomics identify DESI2 as a novel component of the JAK2‐V617F complex, which associates with and stabilizes mutant JAK2 through deSUMOylation and deubiquitination, therefore promoting JAK2 mutant cell growth and MPN disease onset in vivo. Chemical genetics screening followed by structure optimization discovers WWQ‐03‐012, which selectively degrades mutant JAK2 by targeting DESI2, inducing leukemia cell death and inhibiting MPN progression in vitro and in vivo.

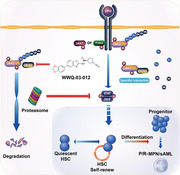

## 1 Introduction

The Janus Kinase 2 (JAK2) plays an essential role in the regulation of hematopoiesis, being essential for signaling by hematopoietic receptors. The JAK2‐V617F mutation, which leads to constitutive kinase activation, is the most common genetic event in myeloproliferative neoplasms (MPN). It is present in over 95% of polycythemia vera (PV), and ≈50% of essential thrombocythemia (ET) and primary myelofibrosis (PMF) cases.^[^ ^1^, ^2^ ^]^ A subset of MPN patients progresses to secondary acute myeloid leukemia (sAML), which is characterized by significant genetic heterogeneity. This heterogeneity renders sAML insensitive to traditional chemotherapy, lacking effective treatment options, and associated with poor prognosis and high relapse rates.^[^ ^3^, ^4^, ^5^ ^]^ Currently, despite advancements in understanding the pathogenesis and the availability of new treatments, such as hematopoietic stem cell transplantation or chemotherapy‐induced remission, the 5‐year overall survival rate for individuals with secondary AML remains ≈12%, while those not receiving such treatments have a 5‐year survival rate of only 1%.^[^ ^6^, ^7^, ^8^, ^9^ ^]^ JAK2 kinase inhibitors, such as Ruxolitinib, show clinical benefit as a monotherapy or in combination with chemotherapy. However, their clinical utility is limited by toxicity to normal cells due to the inhibition of wild‐type (WT) JAK2. Additionally, chronic treatment with JAK2 kinase inhibitors can lead to JAK2 persistent cells, attributed to reactivation of the JAK2 pathway through JAK2‐dependent heterodimeric complexes, which are resistant to degradation and result in the accumulation of phospho‐JAK2.^[^ ^10^, ^11^ ^]^ Therefore, developing a new strategy to target mutated JAK2 for degradation, using the cell's intracellular degradation machinery could provide greater clinical benefits for patients.

The most important intracellular degradation machinery in the cell is the ubiquitin‐proteasome system (UPS), which includes E1 (ubiquitin‐activating enzyme), mediating an ATP‐dependent reaction, E2 (ubiquitin‐conjugating enzyme), and E3 (ubiquitin‐protein ligase), is responsible for degrading most intracellular, soluble proteins, as well as some transmembrane proteins through conjugating and elongating the ubiquitin chains to lysine residues of target proteins.^[^ ^12^, ^13^ ^]^ Similar to ubiquitination, SUMOylation is a posttranslational modification, which involves the addition of SUMO (small Ub‐like modifier) to the lysine residues of numerous proteins, mainly through a two‐step enzyme‐dependent reaction. It is a reversible process analogous to ubiquitylation. The consecutive actions of E1, E2, and E3 enzymes catalyze the attachment of SUMO to target proteins, while deconjugation is promoted by SUMO specific proteases. Recently, it has been discovered that SUMOylation itself can function as a secondary signal mediating ubiquitin‐dependent degradation by the proteasome, whereas SUMO modification can also act as a signal for polyubiquitylation and proteasomal degradation through interplaying or interlinking with each other.^[^ ^14^, ^15^ ^]^ The discovery of ubiquitin ligases bearing SUMO interaction motifs implicated the ubiquitin system in the control of SUMO modified proteins. Besides SUMO specific peptidases (SENPs), DeSUMOylating Isopeptidases (DESIs) are the second class of SUMO proteases, which are reported to control SUMOylation, ubiquitylation, and subcellular localization of target proteins.^[^ ^16^, ^17^ ^]^ Specifically, DESI2 has been demonstrated to enable both Lys48‐ and Lys63‐specific deubiquitinase activity, exerting effects by inhibiting cancer cells growth through decreasing HIF‐1α‐mediated glycolysis or regulating PI3K/AKT/mTOR signaling pathway,^[^ ^18^, ^19^ ^]^ thus presenting a generally complicated picture of this enzyme's role in cancer.

The stability of JAK2 is regulated by both ubiquitination and SUMOylation. Studies have uncovered that suppressor of cytokine signaling 1 (SOCS1) associates with JAK2 phospho‐Y1007 site and inhibits cytokine‐induced JAK2/STAT5 signaling through the ubiquitin‐proteasome pathway.^[^ ^20^ ^]^ The Casitas B‐cell lymphoma (CBL) family E3 ubiquitin ligases downregulate JAK2 stability and signaling via the adaptor protein LNK/SH2B3 in hematopoietic stem cells and myeloid malignancies, while promoting GM‐CSF‐induced full JAK2 activation via K63‐conjugated poly‐ubiquitination at the K970 site in hematopoietic cells.^[^ ^21^, ^22^ ^]^ A recent study reported that WP1130, a promiscuous DUB inhibitor, can destabilize pan‐JAK2 through blocking one of its targets, USP9X.^[^ ^23^, ^24^ ^]^ We also recently found that a deubiquitinase, JOSD1, selectively stabilizes mutant JAK2, and that consequently, compound XL‐106C, a JOSD1 inhibitor, can induce the degradation of mutant JAK2. However, XL‐106C is not suitable for in vivo use because of poor pharmacokinetic properties, further med‐chem efforts are needed to improve the bioavailability of our compounds.^[^ ^25^ ^]^ In addition, JAK2 SUMOylation is required for downstream STAT3 phosphorylation, and the protease SENP1 directly interacts with and deSUMOylates JAK2, leading to its accumulation at cytoplasm and activation of JAK2 in platinum‐resistant ovarian cancer. Thus, blocking SENP1 with inhibitors consequently overcome the resistance.^[^ ^26^, ^27^ ^]^ Therefore, based on evidence that ubiquitination and SUMOylation of JAK2 are related to both its activation and stability, along with the hyperactivation of JAK2 post‐chronic treatment dependent on both the hetero‐dimerization and phosphorylation of its protein, and combined with our recent report of E3 ligands or DUB inhibitors that selectively degrade mutant FLT3, SYK, JAK2 and hyperactivated EZH2,^[^ ^25^, ^28^, ^29^, ^30^, ^31^ ^]^ we proposed to identify novel bifunctional regulators and highly potent inhibitors that enable potential degradation of JAK2‐V617F for MPN or sAML, while also providing potential approaches to treat Ruxolitinib‐resistant patients.

Here, through a mass spectrometry‐based proteomics approach, we identify the deSUMOylases DESI2 as a novel part of the JAK2‐V617F complex. Mechanistically, DESI2 stabilizes JAK2‐V617F through deSUMOylation and deubiquitination of its protein at K962. Moreover, genetic depletion of DESI2 suppresses both JAK2 mutant leukemia cell growth and MPN disease onset in vitro and in vivo. Importantly, DESI2 protein is specifically and highly expressed in JAK2‐mutant cell lines and MPN primary clinical samples, revealed that DESI2 is an unfavorable prognostic marker in MPN, suggesting that targeting DESI2 could be a novel effective therapy. Furthermore, through a compound library screening, followed by chemical proteomics profiling and structure optimization, we discovered WWQ‐03‐012 as a potent inhibitor of DESI2 enzymatic activity (IC_50_ = 47.3 nm). WWQ‐03‐012 can selectively degrade mutant JAK2 and significantly inhibit the tumor growth and MPN progression in vivo across various models. Moreover, its ability to overcome Ruxolitinib resistance and demonstrate synergistic effects when combined with Ruxolitinib further underscores its potential as an effective strategy to enhance therapeutic responses in JAK2‐mutant malignancies. Taken together, our studies provide a novel therapeutic target and an alternative approach for MPN and sAML through targeting mutated JAK2 signaling.

## 2 Results

### 2.1 Mass Spectrometry Analysis Identifies DESI2 as a Novel Component of the JAK2‐V617F Complex

Given that cells persist after chronic inhibitor treatment while remaining JAK2‐dependent, and that the ubiquitination and SUMOylation of JAK2 are closely linked to its activation and stability,^[^ ^20^, ^21^, ^25^, ^26^ ^]^ we aimed to identify novel bifunctional regulators and high potent compounds capable of degrading JAK2‐V617F for the treatment of MPN and sAML. In a prior study, we identified JOSD1, a novel deubiquitinase (DUB) that selectively stabilizes mutant JAK2 through deubiquitination.^[^ ^25^ ^]^ Building on this, efforts to improve the bioavailability of small molecules have led to the identification of additional therapeutic targets and potent compounds.

A schematic overview of the research workflow is provided in Figure S1a (Supporting Information), summarizing the entire experimental process—from the identification of DESI2 as a critical mutation‐specific stabilizer of JAK2‐V617F, through the mechanistic validation of its dual deSUMOylase/deubiquitinase activity, to the development and preclinical evaluation of DESI2 and its inhibitor, WWQ‐03‐012.

**Figure 1:**
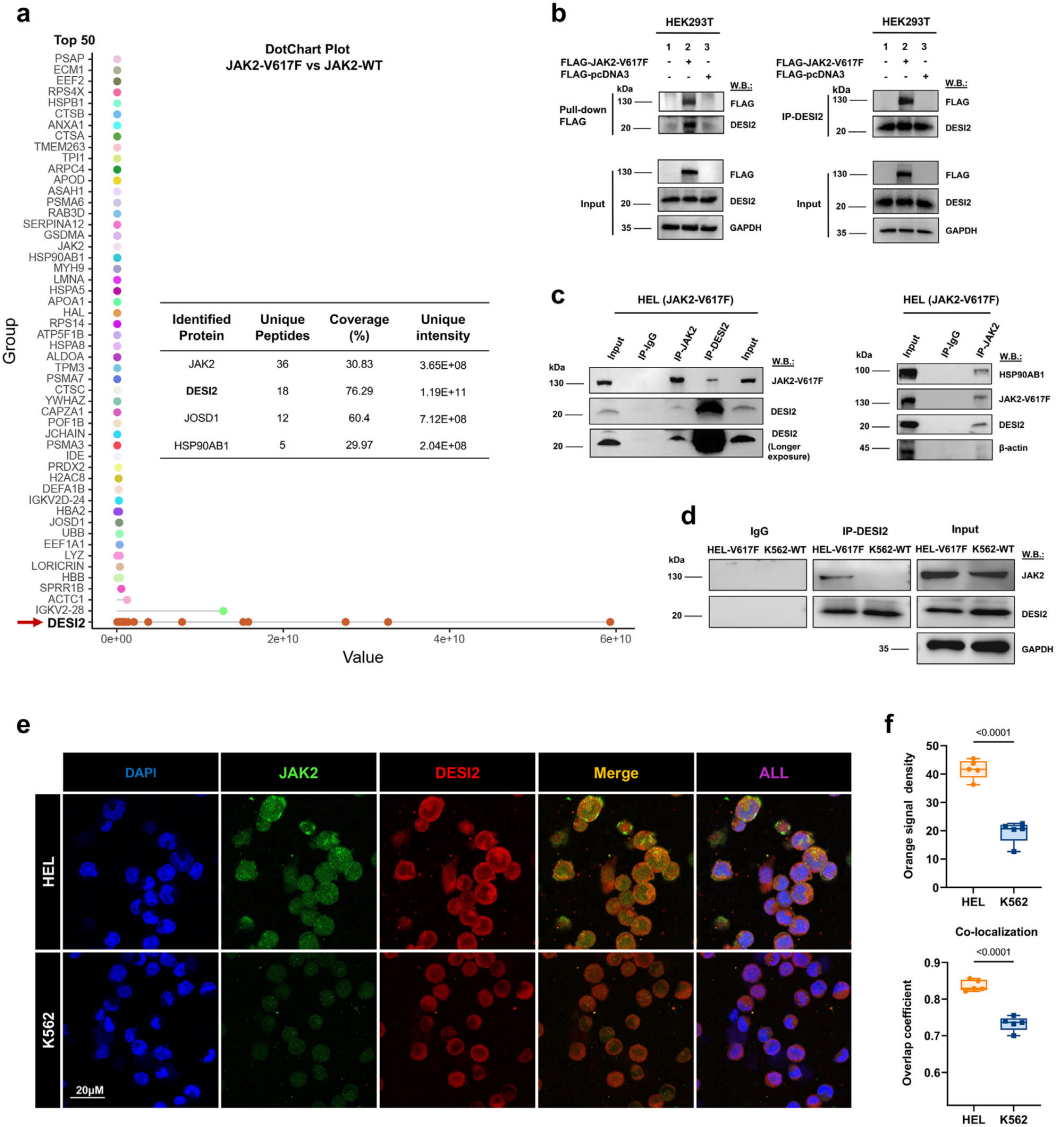
Mass spectrometry analysis identifies DESI2 as a novel component of the JAK2‐V617F complex. a) HEL (JAK2‐V617F) and K562 (JAK2‐WT) cells were collected, and co‐immunoprecipitation (co‐IP) was performed using IgG or anti‐JAK2 antibody, followed by LC‐MS analysis. Proteins potentially forming a complex with JAK2‐V617F are shown (Top 50). Newly identified and previously reported JAK2‐interacting proteins are listed in the right panel. Each dot represents a unique peptide identified by mass spectrometry, with the *x*‐axis indicating peptide intensity. b) Reciprocal IP was conducted using FLAG IgG beads or an anti‐DESI2 antibody in HEK293T cells overexpressing FLAG‐JAK2‐V617F. Both JAK2‐V617F and DESI2 were detected in the immunoprecipitate (IP) and cell lysate (Input) via western blotting with the specified antibodies. c) Reciprocal IP was performed using anti‐JAK2 or anti‐DESI2 antibodies in HEL (JAK2‐V617F) cells. The indicated proteins were detected in the IP and cell lysate (Input) by western blotting with the specified antibodies. d) An IP assay was performed using an anti‐DESI2 antibody in HEL (JAK2‐V617F) and K562 (JAK2‐WT) cells. Both JAK2 and DESI2 were detected in the IP and cell lysate (Input) by western blotting with the specified antibodies. e) HEL (JAK2‐V617F) and K562 (JAK2‐WT) cells were incubated on poly‐L‐lysine‐coated microscope slides. Adherent cells were fixed with paraformaldehyde (PFA) for 30 min, blocked with 1% BSA for 1 h, and then incubated with the specified antibodies. Representative confocal microscopy images (60x) display endogenous DESI2 (red) and JAK2 (green). Cell nuclei were visualized using DAPI (blue); scale bar: 20 µm. f) Objective quantification of co‐localization was performed using ImageJ (upper panel) and Image Pro Plus (lower panel) for colocalization analysis. Shown are the representative results of three independent experiments (n = 3). Error bars represent the mean ± SD. Student's t‐test.

To characterize novel molecules that interact with mutant JAK2, we employed a combination of co‐immunoprecipitation and mass spectrometry analysis. Both K562 wild‐type (WT) JAK2‐expressing cells and HEL cells expressing mutant JAK2 were lysed, and anti‐JAK2 or IgG immunoprecipitation was performed to recover JAK2 molecules along with their associated factors (the JAK2 complex). Mass spectrometry analysis of peptides specifically associated with mutant JAK2 identified DESI2 as the top candidate within the JAK2‐V617F complex (**Figure** **1**a), alongside JOSD1 and HSP90AB1, which served as positive controls.^[^ ^25^, ^32^ ^]^ However, due to the inherent limitations of mass spectrometry, we acknowledge the possibility of false positives and emphasize that these findings require further experimental validation. To confirm whether DESI2 is a component of the JAK2‐V617F complex, we investigated the physical association between DESI2 and JAK2‐V617F through reciprocal co‐immunoprecipitation (co‐IP). HEK293T cells were transfected with constructs encoding FLAG‐tagged JAK2‐V617F, and the cells were harvested and lysed 48 h post‐transfection. Reciprocal co‐IP was performed with FLAG IgG beads or anti‐DESI2 antibody, along with their interacting partners. Both JAK2‐V617F and DESI2 were detected in the immunoprecipitate (IP) and cell lysate (Input) by immunoblotting with the indicated antibodies, suggesting that these two molecules may form a complex (Figure 1b). To further support the notion that JAK2‐V617F interacts with DESI2, we then performed endogenous co‐IP experiments. Specifically, immunoprecipitation of JAK2‐V617F from the lysate of HEL cells readily pulled down DESI2. Conversely, this deSUMOylases was also able to co‐immunoprecipitate JAK2‐V617F in reciprocal experiments (Figure 1c), with HSP90AB1 serving as a positive control and β‐Actin as a negative control.

To clarify the mutant specificity of the DESI2–JAK2 interaction, we used K562 cells (WT JAK2) and HEL cells (JAK2‐V617F), both of which exhibit comparable levels of DESI2 expression. Co‐IP experiments demonstrated that DESI2 selectively binds to JAK2‐V617F (Figure 1d). This finding aligns with the mass spectrometry results and further corroborates the specificity of the interaction with the mutant form (Figure 1a). To further validate that the DESI2–JAK2 interaction is specifically driven by the JAK2‐V617F mutation rather than by differences in cellular background, we performed two orthogonal and isogenic experiments. First, we utilized Ba/F3‐wt and Ba/F3‐JAK2‐V617F isogenic cell lines and performed reciprocal co‐immunoprecipitation (Co‐IP) assays using DESI2 antibodies. To ensure that the enhanced binding was not simply due to higher DESI2 expression, DESI2 protein levels were quantified and normalized to comparable levels between the two lines. The results demonstrated that DESI2 specifically interacted with JAK2‐V617F, but not with wild‐type JAK2, within the isogenic background (Figure S1b, Supporting Information). Second, in the same human K562 cell background, we expressed exogenous JAK2‐WT or JAK2‐V617F using identical pcDNA4.1 constructs under the same promoter to ensure comparable expression levels. Consistent with the isogenic model, Co‐IP assays confirmed a unique DESI2–JAK2 interaction in JAK2‐V617F–expressing cells (Figure S1c, Supporting Information). Collectively, these results establish that DESI2 binding is mutation‐dependent and not confounded by cellular or genetic background differences.

We also performed immunofluorescence staining to assess the co‐localization of DESI2 with both WT and JAK2‐V617F, using colocalization analysis with ImageJ and Image Pro Plus. The results revealed a stronger co‐localization of DESI2 with JAK2‐V617F compared to WT JAK2, which is consistent with our findings from the Co‐IP experiments (Figure 1e,f). Specifically, in HEL cells, JAK2 is potentially subjected to deSUMOylation and deubiquitination through its specific interaction with DESI2, leading to increased cytoplasmic accumulation. In contrast, in K562 cells, JAK2 predominantly localizes within the nucleus, likely existing primarily in a SUMOylated and ubiquitinated state.^[^ ^24^ ^]^ This distinction suggests that DESI2 plays a critical role in regulating the subcellular localization and post‐translational modifications of JAK2, which may further influence its functional activity in different cellular contexts. Taken together, these data suggest that DESI2 interacts with mutant JAK2 in human AML cells, highlighting a potential molecular mechanism underlying the stability and function of JAK2‐V617F in leukemia.

### 2.2 DESI2 Stabilizes JAK2‐V617F Through deSUMOylation and Deubiquitination

**Figure 2:**
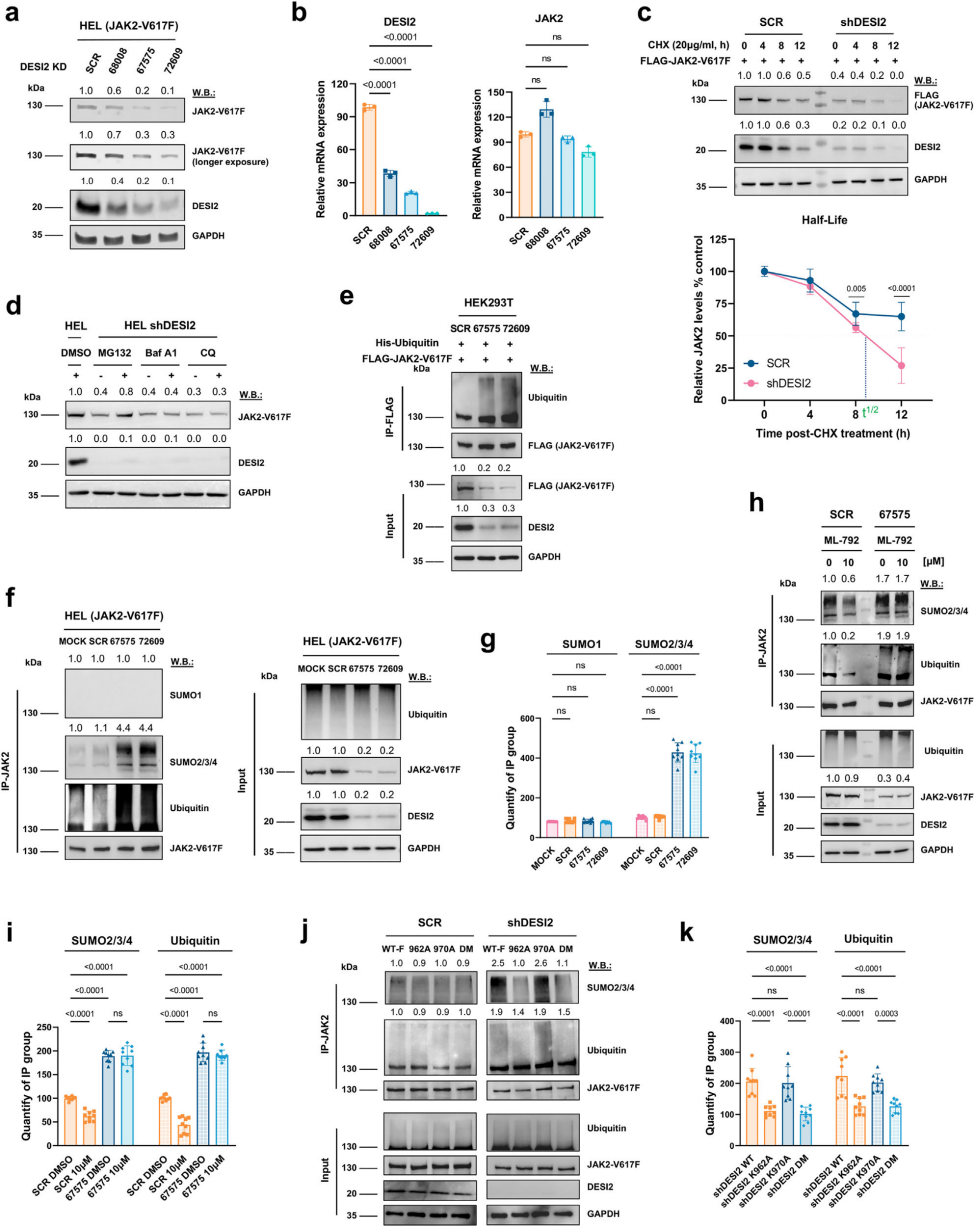
DESI2 stabilizes JAK2‐V617F via deSUMOylation and deubiquitination. a) HEL (JAK2‐V617F) cells were infected with lentiviruses expressing DESI2‐targeting shRNAs (68008, 67575, 72609) or a scrambled control (SCR), followed by puromycin selection for 7 days to establish stable DESI2 knockdown. Protein levels of JAK2‐V617F, DESI2, and GAPDH were analyzed by western blotting using the indicated antibodies. b) The mRNA levels of JAK2‐V617F and DESI2 were quantified by qPCR for the samples in panel (a). Error bars represent the mean ± SD. Student's t‐test. c) HEK293T cells were infected with shRNA‐expressing lentiviruses and selected with puromycin for 7 days, followed by transfection with plasmids encoding JAK2‐V617F. Cycloheximide (CHX, 20 µg mL^−1^) was added, and cells were harvested at the indicated time points. JAK2‐V617F levels and relative turnover rates were determined via immunoblotting with the specified antibodies. Shown are the representative results of three independent experiments (left panel). Error bars represent the mean ± SD. Student's t‐test. The half‐life curves (right panel) were generated by combining the quantitative results from all three independent experiments. d) Cells were prepared as described in panel (a) using shDESI2 (72609), followed by treatment with or without the proteasome inhibitor MG132 (5 µm, 6 h) or the lysosomal inhibitors Bafilomycin A1 (50 µm, 12 h) or Chloroquine (30 µm, 12 h). Protein levels of JAK2‐V617F and GAPDH were subsequently analyzed by western blotting using the indicated antibodies. e) The effect of DESI2 knockdown on JAK2‐V617F ubiquitination was assessed. Cells prepared as described in panel (c) were transfected with JAK2‐V617F and His‐ubiquitin expression plasmids, followed by treatment with 20 µm MG132 for 4 h prior to harvest. Ubiquitinated JAK2‐V617F proteins were visualized after pull‐down with FLAG‐beads. f,g) Cells prepared as described in panel (d) were used to detect SUMOylated or ubiquitinated JAK2‐V617F proteins via pull‐down with JAK2 antibody. Error bars represent the mean ± SD. The ordinary one‐way ANOVA. h,i) Cells prepared as described in panel (a) and treated with or without ML‐792 (a SUMO‐activating enzyme inhibitor) for 24 h. SUMOylated and ubiquitinated JAK2‐V617F proteins were detected using the indicated antibodies following pull‐down with JAK2 antibody. Error bars represent the mean ± SD. The ordinary one‐way ANOVA. j,k) Cells prepared as described in panel (c) were transfected with plasmids encoding either the parental JAK2‐V617F (WT‐F) or site‐directed JAK2‐V617F mutants, including a double mutant (DM), for 24 h. Ubiquitinated or SUMOylated JAK2‐V617F was detected using the indicated antibodies following pull‐down with JAK2 antibody. The immunoblot quantifications were performed using ImageJ software. Shown are the representative results of three independent experiments (n = 3). Error bars represent the mean ± SD. The ordinary one‐way ANOVA.

As DESI2 exhibits both deSUMOylating and deubiquitinating activity,^[^ ^16^, ^18^ ^]^ we hypothesized that DESI2 may target JAK2‐V617F for stabilization. To test this notion, we introduced DESI2‐silencing shRNA constructs into HEL (JAK2‐V617F) cells via lentiviral transduction. As expected, shRNA‐mediated knockdown (KD) of DESI2 reduced JAK2‐V617F protein levels, while the scrambled control (SCR) construct had no effect (**Figure** **2**a). Notably, there was no significant reduction in JAK2 mRNA levels following DESI2 knockdown (Figure 2b). Immunostaining and fluorescent microscopy in JAK2‐V617F‐EGFP‐expressing cells further confirmed the destabilization of JAK2‐V617F upon DESI2 depletion, whereas cells with normal DESI2 expression showed no such effect (Figure S2a, Supporting Information). Taken together, these findings suggest that the loss of DESI2 leads to destabilization of JAK2‐V617F at the protein level, without affecting gene transcription.

We next examined the impact of genetic depletion of DESI2 on the half‐life of JAK2‐V617F. Cycloheximide (CHX) treatment of HEK 293T cells expressing FLAG‐tagged JAK2‐V617F resulted in a reduction of JAK2‐V617F levels at 8 and 12 h post‐treatment. Notably, DESI2 KD significantly shortened the JAK2 protein half‐life to ≈9 h in this cell line (Figure 2c; Figure S2b, Supporting Information). To exclude off‐target effects, we ectopically expressed DESI2 in DESI2 depleted cells and found that expression of DESI2 significantly restored the protein levels of mutant JAK2, but not those of JAK2 WT, in DESI2 KD cells (Figure S2c, Supporting Information). Importantly, DESI2 knockdown in JAK2‐WT‐expressing cells, followed by ectopic re‐expression, showed little to no effect on WT JAK2, suggesting that DESI2 preferentially targets mutant JAK2 over WT JAK2 (Figure S2d,e, Supporting Information). To further determine whether this effect depends on its enzymatic activity, we performed rescue experiments in HEL cells using 3′ UTR–targeting hairpins for DESI2 knockdown, followed by reintroduction of either WT DESI2 or the catalytically inactive (Cys108→Ser) mutant. Only WT DESI2 restored JAK2‐V617F stability, underscoring the essential role of DESI2's catalytic activity (Figure S2f, Supporting Information). Moreover, DESI2 depletion‐mediated degradation of mutant JAK2 was rescued by proteasome inhibition with MG132 in endogenous systems using different hairpins, but not by lysosome inhibitors (Bafilomycin A1 or Chloroquine) (Figure 2d; Figure S2g, Supporting Information). This suggests that DESI2 regulates the ubiquitin‐mediated proteasomal degradation of mutant JAK2. We then employed a co‐IP approach to further investigate this mechanism and observed increased JAK2‐V617F ubiquitination following DESI2 KD. Specifically, DESI2 KD using each hairpin resulted in a robust increase in poly‐ubiquitination levels, along with substantial degradation of JAK2‐V617F, compared to the SCR control (Figure 2e). These results strongly support the notion that DESI2 stabilizes JAK2‐V617F by interacting with and deubiquitinating it.

To further investigate whether DESI2 mediates the SUMOylation of JAK2‐V617F, we again performed IP in DESI2 KD lines, and the different types and levels of SUMOylation were evaluated by immunoblotting. Similarly, KD of DESI2 in these lines increased SUMOylated JAK2‐V617F (SUMO2/3/4, while not SUMO‐1‐linked), compared to shRNA vector control treatment. Simultaneously, DESI2 knockdown resulted in a significant increase in JAK2‐V617F ubiquitination (Figure 2f,g; Figure S3a, Supporting Information). The discrepancy between the IP and lysate lanes may be attributed to the immunoprecipitation reaction reaching saturation, where the antibody becomes the limiting reagent, a phenomenon that has been documented in similar studies.^[^ ^33^, ^34^ ^]^ In order to determine whether DESI2 affects JAK2‐V617F ubiquitination through deSUMOylation, we treated DESI2‐depleted cells with ML‐792, a potent inhibitor of the SUMO‐activating enzyme. We observed that ML‐792 treatment decreased the SUMOylation of JAK2‐V617F, followed by a reduction in ubiquitination levels. However, this effect was not observed in DESI2 knockdown cells (Figure 2h,i), suggesting that DESI2's deSUMOylation activity is essential for its removal of the ubiquitination from mutant JAK2.

We then investigated the deSUMOylation/deubiquitination sites by analyzing co‐IP samples using liquid chromatography‐mass spectrometry (LC‐MS). Combined with analysis and prediction by the SUMOplot Analysis Program, we identified two lysines, K962 and K970, as candidate SUMOylation/ubiquitination sites on JAK2‐V617F. We then generated lysine site or double mutations (K962A, K970A, or DM (both K to A)) in JAK2‐V617F‐kinase domain, and overexpressed them in HEK293T DESI2‐KD cells. The SUMOylation/ubiquitination sites were visualized and confirmed through ubiquitination assays. The results showed that only K962 is critical for DESI2‐mediated deSUMOylation/deubiquitination of JAK2‐V617F within the kinase domain (Figure S3b–d, Supporting Information; Figure 2j,k). We also assessed JAK2‐V617F‐K962A levels following DESI2 KD through ubiquitination assays. The results showed that the K962A mutation prevents DESI2‐mediated deubiquitination of JAK2‐V617F, rendering it resistant to degradation after DESI2 KD for 72 h (Figure S3e, Supporting Information). We further co‐transfected JAK2‐V617F‐K962A and catalytic‐dead DESI2 (Cys108→Ser) mutants and performed co‐IP experiments to assess their interaction. The results demonstrated that both mutations significantly disrupted DESI2–JAK2‐V617F binding and stability, confirming that the binding and functional activity of DESI2 are critically dependent on these sites (Figure S3f, Supporting Information).

To further validate these observations, we utilized AlphaFold3^[^ ^35^ ^]^ to predict the binding modes of DESI2 with both WT and JAK2‐V617F. The modeling revealed that the JAK2‐V617F mutation induces partial activation, resulting in significant conformational changes, which are consistent with previously reported findings,^[^ ^36^, ^37^, ^38^ ^]^ particularly in the FERM and JH1 regions, which are exposed and form multiple strong interactions with DESI2 (a total of seven hydrogen bonds). In contrast, the JH1 domain of WT JAK2 is obstructed by the JH2 and SH2 regions, which results in a less stable and weaker interaction with DESI2 (three fewer hydrogen bonds compared to the mutant), limited to the FREM region. More importantly, this binding mode places the enzymatic active site of DESI2, Cystine 108, in close proximity to the ubiquitination/SUMOylation site (K962) on JAK2‐V617F, which we confirmed through mass spectrometry and ubiquitin‐pulldown assays. In contrast, for WT JAK2, DESI2 is positioned significantly farther from these modification sites, reducing the likelihood of interaction (Figure S4, Supporting Information). This binding mode likely explains how DESI2 selectively binds to mutant JAK2 and stabilizes it through deSUMOylation and deubiquitination at the K962 site. This mechanism provides a logical basis for the mutant‐specific action of DESI2, shedding light on the driving force behind its selective interaction with the mutant form.

### 2.3 DESI2 is Highly Expressed in JAK2‐Mutant Cells and Depletion of DESI2 Suppress JAK2 Mutant Cell Growth

**Figure 3:**
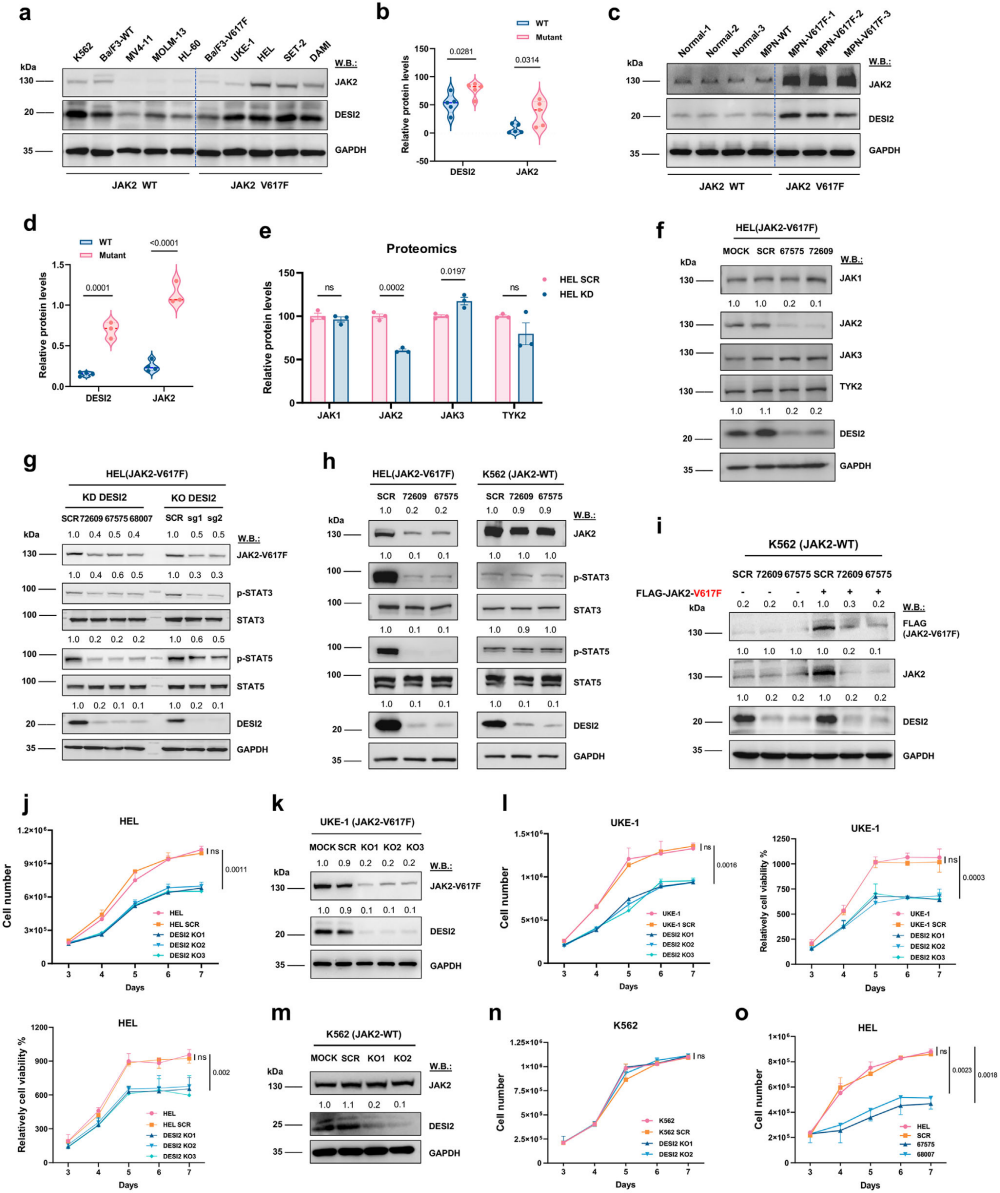
DESI2 is highly expressed in JAK2‐mutant cells and its depletion suppresses their growth in vitro. a,b) AML cells expressing either wild‐type JAK2 or JAK2‐V617F (n = 5) were collected for protein‐level analysis. The protein levels of JAK2, DESI2, and GAPDH were analyzed by immunoblotting with the indicated antibodies. Protein expression levels were quantified using ImageJ by measuring the integrated optical density (IOD) of each band. To ensure comparability, all samples within the same blot were normalized to the corresponding GAPDH signal. The relative expression values are presented as the mean ± SD from five independent cell lines, with all experiments conducted under identical exposure and detection conditions. Student's t‐test. c,d) Normal bone marrow and primary MPN patient samples expressing wild‐type JAK2 or JAK2‐V617F (n = 3 or 4) were collected for protein expression analysis. The protein levels of JAK2, DESI2, and GAPDH were analyzed by immunoblotting with the indicated antibodies. Protein expression levels were quantified as described for Figure 3b, using densitometric analysis. Data are shown as mean ± SD. Student's t‐test. e,f) Proteomic and immunoblotting analyses were performed to evaluate JAK family protein levels following DESI2 knockdown in HEL (JAK2‐V617F) cells. Data are shown as mean ± SD. Student's t‐test. g) DESI2 was knocked down using various shRNA hairpins or knocked out using the CRISPR‐Cas9 system in HEL (JAK2‐V617F) cells. The effects of DESI2 KD or KO on JAK2 protein levels and downstream signaling in JAK2‐V617F‐positive HEL cells were assessed. h) The impact of DESI2 KD on WT JAK2 (K562) and JAK2‐V617F (HEL) protein levels and downstream signaling, was assessed by western blotting with the indicated antibodies. i) DESI2‐knockdown K562 cells were simultaneously transfected with FLAG‐tagged JAK2‐V617F (pcDNA4.1 under identical promoters). Immunoblotting was performed for FLAG, JAK2, DESI2, and GAPDH. j‐l) The impact of DESI2 KO on the growth and viability of JAK2‐V617F‐positive HEL and UKE‐1 cells was evaluated through growth and cell viability assays. m,n) The impact of DESI2 KO on the growth of JAK2‐WT K562 cells was assessed through growth assays. o) The impact of DESI2 KD on the growth of JAK2‐V617F^+^ HEL cells was assessed through growth assays. Shown are the representative results of three independent experiments (n = 3). Data are shown as mean ± SD. The ordinary one‐way ANOVA.

To determine the functional consequence of the DESI2‐JAK2 complex, we first assessed whether DESI2 could serve as a clinical biomarker for AML. To this end, we first collected AML cells expressing either WT JAK2 or JAK2‐V617F (n = 5) for protein‐level analysis. The results clearly demonstrated a significant upregulation of DESI2 protein levels in JAK2‐mutant‐driven cell lines, highlighting its specific correlation with the stabilization of the mutant JAK2 protein (**Figure** **3**a,b). Additionally, we analyzed normal bone marrow and primary MPN patient samples expressing wild‐type JAK2 or JAK2‐V617F (n = 3 or 4), confirming the consistent findings observed in cell line models, with DESI2 expression significantly higher in JAK2‐V617F mutant samples (Figure 3c,d). This finding further underscores DESI2's potential as a specific therapeutic target for JAK2‐mutant AML/MPN.

To investigate the functional consequences of DESI2 depletion, we performed proteomic analysis following DESI2 KD. This analysis identified JAK2‐V617F as a key protein affected by DESI2, reinforcing the main conclusions of our study. Notably, DESI2 KD did not decrease the protein levels of other JAK family members, demonstrating its specificity for JAK2, as confirmed by western blotting (Figure 3e,f). Further genetic depletion studies in HEL and UKE‐1 cells showed significant reductions in JAK2‐V617F protein levels and downstream signaling in DESI2 KD/KO cells compared to SCR controls. Notably, there was little to no impact on the expression of molecules downstream of JAK2, including STAT3, STAT5, AKT, and ERK/MAPK (Figure 3g; Figure S5a,b, Supporting Information). Specifically, we knocked down DESI2 in both K562 (JAK2‐WT) and HEL (JAK2‐V617F) cells, results shown that DESI2 loss inducing a significant degradation of JAK2‐V617F and downstream signaling, while little to no effect on wide‐type (WT) JAK2 (Figure 3h), which is consistent with our previous results (Figure 1d,e; Figures S1b,c and S2c–e, Supporting Information). To further confirm the specificity of DESI2 in stabilizing JAK2‐V617F, we conducted both forward and reverse validation experiments. 1) Forward Validation: We overexpressed FLAG‐tagged JAK2‐V617F in K562 (JAK2‐WT) cells, performed DESI2 knockdown (KD) simultaneously, and examined the expression changes of both wild‐type and mutant JAK2. The results clearly demonstrated that DESI2 knockdown selectively induced the degradation of overexpressed JAK2‐V617F, whereas the endogenous JAK2‐WT in K562 cells remained unaffected, underscoring the specific role of DESI2 in stabilizing the mutant protein (Figure 3i). 2) Reverse Validation: We overexpressed FLAG‐tagged JAK2‐WT in HEL (JAK2‐V617F) cells, performed DESI2 knockdown, and examined the expression changes of both wild‐type and mutant JAK2. In this case, DESI2 knockdown did not affect the expression of JAK2‐WT in HEL cells, providing further evidence that DESI2 specifically stabilizes JAK2‐V617F rather than wild‐type JAK2 (Figure S5c, Supporting Information). We next assessed the impact of DESI2 depletion on AML cell growth. Growth studies in JAK2‐V617F‐positive cell lines (HEL and UKE‐1) and wild‐type JAK2 cells (K562) revealed that DESI2 KD or KO robustly inhibited cell proliferation by promoting the degradation of mutant JAK2 (Figure 3j–o; Figure S5d, Supporting Information). Furthermore, cell viability assays and flow cytometry analysis of apoptosis signaling showed that DESI2 KO/KD significantly reduced cell viability and induced apoptosis (Figure 3j,l; Figure S5e, Supporting Information). These findings suggest that the loss of DESI2 destabilizes JAK2‐V617F signaling, thereby compromising cell viability in JAK2‐V617F‐dependent cells. These findings support the notion that DESI2 might serve as a novel therapeutic target for mutant JAK2‐expressing‐AML.

### 2.4 DESI2 Deficiency Impairs JAK2 Mutant AML Progression and Suppresses MPN Disease Onset In Vivo

Given the role of DESI2 in deubiquitinating and stabilizing of JAK2 protein as revealed by our in vitro experiments, we next sought to investigate whether DESI2 contributes to AML and MPN pathogenesis in different in vivo models. We first performed subcutaneous (s. c.) implantation of SCR or DESI2 KD (shDESI2) JAK2‐V617F^+^ HEL cells (1 × 10^7^ cells/mouse; n  =  7 mice/group) into female NOD scid gamma (NSG) mice (6–8 weeks old). Tumor growth was significantly slower, and survival was notably extended in the shDESI2 group compared to the SCR control group, indicating that DESI2 plays a pathological role in promoting disease progression in vivo (Figure S6a–c, Supporting Information).

**Figure 4:**
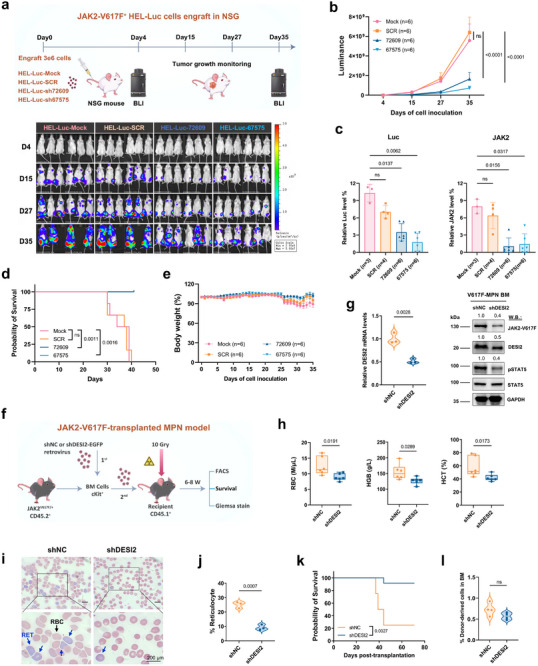
DESI2 deficiency impairs JAK2 mutant AML progression and suppresses MPN disease onset in vivo. a) HEL‐Luc‐GFP cells were first established via transduction with a firefly luciferase‐expressing lentivirus. Subsequently, DESI2 knockdown was achieved using shRNAs (72609, 67575), with SCR and Mock serving as controls. Luminescence signal was confirmed as indicated. Cells were then grown in a tail vein injection‐based non‐invasive in vivo bioluminescence model of leukemia (n = 6). Bioluminescent images of representative mice with matched initial leukemia burden are shown. b) Total flux bioluminescence plotted as a graph. c) Correlation between luciferase‐positive leukemia burden measured by the Bright‐Glo assay and Luminoskan (left panel). The percentage of HEL‐GFP‐Luc and JAK2‐V617F^+^ (GFP^+^JAK2^+^) cells in the bone marrow, as measured by flow cytometry using both GFP (PE) and JAK2‐Alexa Fluor 594 antibody, from bone marrow (BM) samples of Mock, SCR, 72609, and 67575 transplanted mice (n = 3 – 6) (right panel). The term “relative” JAK2 expression levels refers to the percentage of JAK2‐positive cells within the total bone marrow population. d) Kaplan–Meier survival analysis of xenograft mice. e) Body weights of xenograft mice over 35 days. f) Schematic of the strategy for DESI2 silencing effects on JAK2^V617F^‐driven MPN mice (n = 6). g) EGFP‐tagged shDESI2 virus was used to transduce bone marrow cells from JAK2^V617F^‐knockin mice. EGFP‐positive cells (DESI2 KD) were sorted 48 h post‐infection and transplanted into lethally irradiated recipient mice. Bone marrow was harvested four weeks post‐transplantation for analysis by QPCR and immunoblotting. h) Statistical analysis of red blood cells (RBC), hemoglobin (HGB), and hematocrit (HCT) counts in peripheral blood from the indicated groups of mice as in (f) after treatment. shNC represents a non‐targeting shRNA. Each dot represents one mouse. i) Representative peripheral blood smear (PBS) of indicated groups of mice, as in (f). Typical RBCs and reticulocytes (RET) are indicated. Scale bar = 200 µm. j) The proportion of immature reticulocytes in the peripheral blood of mice was determined. k) Kaplan–Meier survival analysis of transplanted mice (n = 8). l) c‐Kit^+^ cells from JAK2^V617F+^ mouse bone marrow were infected with retroviruses encoding shDESI2 and transplanted into lethally irradiated recipients. The homing rate was calculated by measuring the number of LSK^+^GFP^+^ cells in the recipients’ bone marrow 18 h post‐transplantation, relative to the total transplanted cells. Error bars represent the ± SD. Student's t‐test or ordinary one‐way ANOVA, and log‐rank test.

Next, we generated luciferase‐positive HEL (HEL‐Luc^+^GFP^+^, JAK2‐V617F) and K562 (K562‐Luc^+^GFP^+^, JAK2‐WT) cells by transducing a firefly luciferase‐expressing lentivirus, followed by DESI2 depletion (Figures S6d and S7a, Supporting Information). These cells were used for tail vein non‐invasive implantation studies in female NSG mice (n = 6/group). As expected, DESI2 depletion significantly reduced leukemia burden in HEL‐Luc^+^ mice compared to SCR or mock controls, as shown by in vivo bioluminescence imaging (**Figure** **4**a,b). In contrast, no significant effect was observed in K562‐Luc^+^ mice (Figure S7b,c, Supporting Information). After six weeks, mice were sacrificed, and bone marrow samples were collected for analysis. Flow cytometry was conducted to assess the proportion of GFP^+^JAK2‐V617F^+^ cells, and the results were compared to the SCR control group. We observed a marked reduction in JAK2‐V617F expression levels in the DESI2‐KD mice, with an ≈3–4‐fold reduction in both luciferase signal and JAK2‐V617F levels in bone marrow samples from DESI2‐KD mice compared to controls (Figure 4c). Furthermore, DESI2 loss not only reduced tumor burden but also significantly improved survival in JAK2‐V617F‐transplanted mice, with no effect in JAK2‐WT mice (Figure 4d; Figure S7d, Supporting Information), underscoring its therapeutic potential in targeting JAK2 mutant cells in vivo. Importantly, no significant difference in body weight was observed between DESI2‐deficient and control mice over 35–42‐days, suggesting no overt toxicity (Figure 4e; Figure S7e, Supporting Information). Taken together, these findings demonstrate that DESI2 deficiency effectively impairs JAK2 mutant AML progression in vivo, highlighting its critical role in the expansion of JAK2 mutant cells, making it a promising target for therapeutic intervention.

We next examined the in vivo role of DESI2 in the onset of MPN disease. Bone marrow mononuclear cells purified from JAK2^V617F^‐driven MPN mice,^[^ ^39^ ^]^ followed by depletion of DESI2 using EGFP^+^ shDESI2. The EGFP^+^ cells were then sorted and immediately transplanted into lethally irradiated recipient mice (n = 6/group) (Figure 4f). The successful depletion of DESI2 was confirmed by QPCR and immunoblotting of total bone marrow cells from recipient mice 1 month after transplantation (Figure 4g). Two months post‐transplantation, the red blood cells (RBC), hemoglobin (HGB), and hematocrit (HCT) indices (in millions [M] of cells per µL) of indicated mice were analyzed. Results showed that mice with DESI2 knockdown exhibited significant lower RBC, HGB, and HCT counts in peripheral blood from the indicated groups of mice, indicating that DESI2 may inhibit MPN onset (Figure 4h). Furthermore, the proportion of immature reticulocytes in the peripheral blood was significantly lowered in DESI2 KD mice, as demonstrated by Giemsa stain of peripheral blood smears (PBS), suggesting DESI2 KD hinders the onset of JAK2^V617F^‐driven MPN disease (Figure 4i,j). To evaluate the long‐term impact of DESI2 knockdown, survival tracking was extended to 10 weeks, revealing that DESI2 knockdown significantly prolonged survival in these mice (Figure 4k). While most JAK2^V617F^ knock‐in mice typically survive beyond 40 days post‐transplantation, our findings are consistent with previous studies, which attribute the lethality of JAK2^V617F^‐driven disease primarily to thrombotic complications rather than classic myelofibrosis in both humans and mice.^[^ ^39^, ^40^, ^41^, ^42^, ^43^ ^]^ Similar to prior reports, we observed clinical signs of thrombosis,^[^ ^44^ ^]^ such as hindlimb paralysis and tachypnea, with necropsy confirming large‐vessel occlusions, particularly in the lungs and kidneys, as the main cause of mortality in these mice.

Additionally, the homing rate of transplanted bone marrow cells following DESI2 knockdown was assessed. We observed that the loss of DESI2 had minimal effect on the homing of HSCs (LSK⁺GFP⁺ cells) 18 h post‐transplantation (Figure 4l). This finding aligns with previous studies showing that while the JAK2‐V617F mutation alters HSC function and proliferation, it has minimal impact on HSC homing.^[^ ^39^ ^]^ Together, these results further underscore DESI2 as a promising therapeutic target for JAK2^V617F^‐driven MPN disease.

### 2.5 Compounds Screening and Structure Optimization Identifies WWQ‐03‐012 as a Selective Degrader of Mutant JAK2 via DESI2 Inhibition

**Figure 5:**
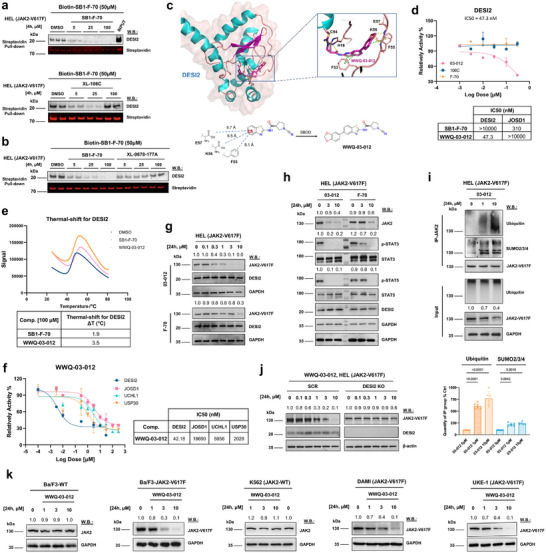
Compound screening and structure optimization identify WWQ‐03‐012 as a selective degrader of mutant JAK2 via DESI2 inhibition. a,b) A chemical genetics screen of a DUB‐focused small molecule inhibitor library identified SB1‐F‐70 as selectively binding DESI2. A competitive activity‐based biotin‐streptavidin pulldown assay was performed: HEL lysates (2 mg per sample) were pretreated with biotinylated SB1‐F‐70 (50 µm) for 4 h, followed by incubation with SB1‐F‐70 or XL‐106C (0, 5, 25, 100 µm). Biotin‐streptavidin pulldown assays visualized the binding effects of small molecules on DESI2 via western blotting. XL‐177A (a specific USP7 inhibitor) served as a negative control. c) Strategic design of potent and selective DESI2 inhibitors: Top panel: Molecular dynamics‐based prediction of WWQ‐03‐012 binding modes to DESI2 protein (AlphaFold‐derived structures for DESI2). Bottom panel: Overview of structure‐based drug design for novel DESI2 inhibitors developed in this study. Interactions are highlighted as H‐bonds (yellow dashes) and π–π interactions (green dashes). d) Dose‐response analysis confirmed that WWQ‐03‐012 inhibits DESI2 in a purified enzyme biochemical assay (Ub‐AMC) with an IC50 of 47.3 nm. e) A thermal shift assay was performed with 100 µm SB1‐F‐70 or WWQ‐03‐012 on DESI2 to evaluate binding. f) IC50 of WWQ‐03‐012 in a purified enzyme biochemical assay (Ub‐Rho) against DESI2, UCHL1, USP30, and JOSD1. g) HEL cells were treated with 03–012 and F‐70 at various concentrations for 24 h. JAK2‐V617F, DESI2, and GAPDH protein levels were analyzed via western blotting. h) HEL cells were treated with 03–012 and F‐70 at 0, 3, and 10 µm for 24 h. Various proteins were assessed by western blotting using the indicated antibodies. i) HEL cells were treated with 03–012 for 24 h at 0, 1, and 10 µm. Immunoprecipitation (IP) was performed using JAK2 antibody. JAK2‐V617F, ubiquitin, SUMO2/3/4, and GAPDH protein levels were assessed by western blotting. j) DESI2 was knocked out in HEL (JAK2‐V617F) cells using the CRISPR‐Cas9 system. Cells were then treated with 03–012 at the indicated concentrations for 24 h. JAK2‐V617F, DESI2, and β‐actin protein levels were analyzed via western blotting using the indicated antibodies. k) Different JAK2‐WT or JAK2‐V617F‐expressing cells were treated with 03–012 at the indicated concentrations for 24 h. Protein levels of JAK2 and GAPDH were analyzed by western blotting using the indicated antibodies. Shown are the representative results of three independent experiments (n = 3). Data are presented as the mean ± SD. The ordinary one‐way ANOVA.

To identify novel therapeutics selectively targeting DESI2, we designed and conducted a Ub‐AMC/Rho based chemical screen using our in‐house small molecule degrader library (comprising 386 compounds). This was followed by confirmation through chemical proteomics assays.^[^ ^45^ ^]^ The screening assay was an activity‐based chemical proteomic assay designed to assess binding affinity to the active site, rather than enzyme activity. During the screening, SB1‐F‐70, a UCHL1 inhibitor,^[^ ^23^ ^]^ was identified as a selectively HIT compound targeting DESI2 (TOP 1), and these findings was further validated using two independent activity‐based chemical proteomic assays (Figure S8a–c, Supporting Information). We then performed a “target‐engagement” assay to determine whether SB1‐F‐70 could label DESI2. To achieve this, we designed and synthesized Biotin‐F‐70, a biotinylated homolog of SB1‐F‐70, and conducted a competition assay using cell lysate treated with Biotin‐F‐70 after 4 h incubation with SB1‐F‐70 in a dose‐dependent manner. This investigation revealed that 25 µm Biotin‐F‐70 effectively labeled ≈50% of DESI2 after 4 h of treatment. In addition, XL‐106C, an analog of SB1‐F‐70 developed to inhibit JOSD1,^[^ ^25^ ^]^ also partially labeled DESI2 (**Figure** **5**a). In contrast, XL‐177A, a selective USP7 inhibitor,^[^ ^46^ ^]^ did not compete with Biotin‐F‐70 for DESI2 binding, as no dose‐dependent decrease in DESI2 was observed (Figure 5b). These results confirm that DESI2 is a direct target of SB1‐F‐70.

Our initial screening identified SB1‐F‐70 as a promising compound based on strong target engagement with DESI2. However, subsequent enzymatic assays revealed weak inhibitory potency (IC50 > 30 µm) (Figure S8a, Supporting Information), prompting us to optimize this scaffold through medicinal chemistry efforts. Building on our previous work, which showed that SB1‐F‐70 had a mild effect on JAK2 degradation through targeting JOSD1, we proceeded to synthesize and optimize a new covalent analog. This led to the development of WWQ‐03‐012, a compound with significantly higher potency toward DESI2, as compared to JOSD1 (Figure 5c,d). Briefly, the lead molecules for the cyanamide‐based DESI2 covalent inhibitors were obtained during the evaluation of several scaffolds, which ultimately led to the discovery of 106C and F‐70. Through docking studies of F‐70 and DESI2, we reasoned that an aromatic ring linked to the C5 position on the benzothiazole ring might form π–π interactions with Phe55 and enhance the binding affinity (Figure 5c). Furthermore, we proposed that extending the aromatic ring further into the binding pocket by introducing an ether linkage would further improve the activity by forming more interactions with Lys56 and Glu57. Considering synthetic feasibility, a 1,3‐benzodioxole ring was linked to the C5 position of the benzothiazole ring, leading to the synthesis of WWQ‐03‐012 (Figure 5c). This compound was optimized for maximal JAK2‐V617F degradation, as assessed by western blotting and densitometry (data not shown). We next performed enzyme activity assays with thiazole‐derived DESI2 inhibitors, including the optimized compound, to evaluate their effect on DESI2 activity and IC_50_ using the Ub‐AMC assay. As expected, a comparison of the activities of WWQ‐03‐012 and F‐70 revealed that the optimization efforts significantly improved activity (Figure 5d; Figure S9a, Supporting Information). Moreover, the MD‐based prediction of binding modes of WWQ‐03‐012 with DESI2 protein demonstrated that the covalent conduct was formed between WWQ‐03‐012 and Cys94 of DESI2, and the optimization initiatives effectively enhanced the interactions between WWQ‐03‐012 and DESI2 (Figure S9b, Supporting Information). To better understand the differences in binding, we conducted a thermo‐shift assay comparing the affinities of SB1‐F‐70 and WWQ‐03‐012 for DESI2, the analysis revealed that F‐70 induced a shift of 1.96 °C, while 03–012 caused a larger shift of 3.5 °C, confirming that WWQ‐03‐012 exhibits significantly stronger binding (Figure 5e).

Since WWQ‐03‐012 was derived from SB1‐F‐70, we evaluated its activity against a selected set of purified DUBs, including UCHL1, USP30, JOSD1, and DESI2. Our results show modest activity against UCHL1 (IC50 = 5956 nm) and USP30 (IC50 = 2028 nm). However, based on our previous findings, UCHL1 is expressed at very low levels in JAK2‐V617F^+^ cell lines (undetectable by Western blot), and treatment with a specific USP30 inhibitor or USP30 knockout did not result in JAK2 degradation.^[^ ^25^ ^]^ Therefore, we conclude that the off‐target activity of WWQ‐03‐012 against UCHL1 and USP30 is unlikely to significantly affect its impact on JAK2‐V617F degradation (Figure 5f).

As anticipated, compared to its analog, WWQ‐03‐012 exerts greater potency in leukemia cells by promoting the degradation of mutant JAK2, thereby blocking the downstream signaling pathway (IC_50_ = 47.3 nm, DC_50_ = 543.6 nm in HEL) (Figure 5g,h; Figure S9c, Supporting Information). In line with the loss of JAK2‐V617F protein resulting from SUMO‐ubiquitin‐dependent degradation following DESI2 genetic depletion (Figure 2e–i; Figure S3a, Supporting Information), we observed the SUMOylation and ubiquitination of JAK2‐V617F following drug treatment. Notably, WWQ‐03‐012 treatment resulted in a robust increase in poly‐ubiquitination levels and substantial degradation of JAK2‐V617F, compared to the untreated control (Figure 5i). To rule out potential off‐target effects, we treated SCR and DESI2 depleted cells with WWQ‐03‐012, results revealed that while depletion of DESI2 significantly reduced JAK2‐V617F levels, there were no further degradation effects on JAK2‐V617F post‐WWQ‐03‐012 treatment (Figure 5j). To further explore the functional impact of DESI2 inhibition, we performed proteomic analyses following treatment with WWQ‐03‐012 or DESI2 knockdown. The overlap results highlighted JAK2‐V617F as a key protein, ranked in the top 5% (11/273) of those significantly affected by DESI2 (Figure S9d, Supporting Information). Notably, DESI2 inhibition did not affect the protein levels of other JAK family members, demonstrating its specificity for JAK2, as confirmed by western blotting (Figure S9e, Supporting Information), consistent with our previous findings (Figure 3e,f). Furthermore, we evaluated the impact of WWQ‐03‐012 on WT and JAK2‐V617F protein levels across various MPN/AML cell lines. WWQ‐03‐012 significantly degraded the mutant protein while having minimal impact on the WT protein, highlighting its mutation‐selective activity (Figure 5k). These findings strongly support the notion that pharmacologically inhibiting DESI2 promotes the ubiquitin‐mediated degradation of JAK2‐V617F, positioning DESI2 as a promising therapeutic target for mutant JAK2 driven leukemias.

### 2.6 Targeting DESI2 Induces JAK2 Mutant Leukemia Cell Death in Preclinical Models

**Figure 6:**
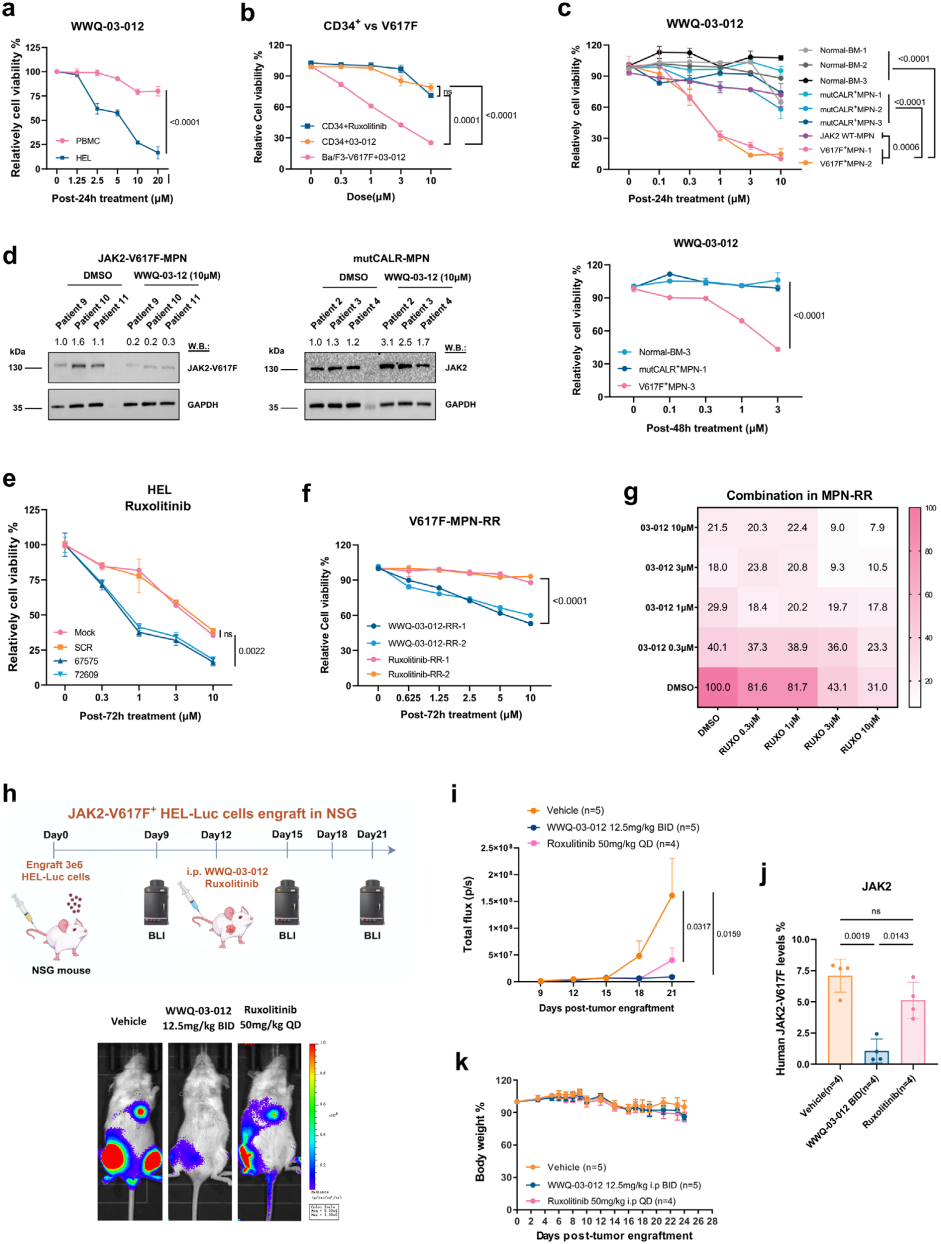
Targeting DESI2 induces JAK2 mutant leukemia cell death in preclinical models. a) Primary human PBMCs and JAK2‐V617F‐positive HEL cells were treated with WWQ‐03‐012 for 24 h, and cell viability was assessed using the CellTiter‐Glo luminescent cell viability assay (CTG). b) Effects of 24 h WWQ‐03‐012 or Ruxolitinib (FDA approved JAK1/2 inhibitor) treatment on mouse normal CD34^+^ cells and Ba/F3‐JAK2‐V617F cells. c) Effects of 24‐ or 48‐h WWQ‐03‐012 treatment on bone marrow samples from healthy donors and MPN patients harboring Calreticulin mutant (mutCALR), JAK2 wild‐type (WT), or JAK2‐V617F mutations (n = 3 per group). d) Immunoblotting: JAK2 protein levels following DESI2 inhibition by WWQ‐03‐012 in JAK2‐V617F and mutCALR‐driven (JAK2 WT) MPN patient samples (n = 3). e) Proliferation Studies: The effects of a 72‐h treatment with or without Ruxolitinib on SCR and DESI2 KD (67575, 72609) HEL cells were assessed. f) Proliferation studies: Effects of 72‐h WWQ‐03‐012 or Ruxolitinib treatment on Ruxolitinib‐resistant/persistent patient samples with JAK2‐V617F. MPN‐RR: Ruxolitinib‐resistant MPN patient. g) Proliferation studies: Assessment of the effects of 24‐h treatment with WWQ‐03‐012, Ruxolitinib, or their combination on Ruxolitinib‐resistant/persistent patient samples with JAK2‐V617F. h) HEL‐Luc cells were generated as shown in Figure 4. The effect of WWQ‐03‐012 on HEL‐Luc^+^ cell growth was assessed in a tail vein non‐invasive in vivo bioluminescence model of leukemia (n = 4–5). Bioluminescent images of representative mice with matched initial leukemia burden. i) Total flux bioluminescence was plotted as a graph. j) The percentage of GFP^+^JAK2^+^ cells in bone marrow samples from vehicle‐ or 03‐012‐, Ruxolitinib‐treated mice (n = 4–5) was measured using flow cytometry with both GFP (PE) and JAK2‐Alexa Fluor 594 antibody. k) Body weights of xenograft mice were monitored for up to 24 days. Shown are the representative results of three independent experiments (n = 3). Data are presented as the mean ± SD. The ordinary one‐way ANOVA.

We next evaluated the therapeutic potential of our lead DESI2 inhibitor, WWQ‐03‐012, by testing its growth inhibitory effect on various preclinical models. First, we compared the inhibitory effects of WWQ‐03‐012 on both WT and JAK2‐V617F‐mutated MPN/AML cell lines (n = 4/group) using a cell viability assay. The results revealed a significant difference in the Growth Inhibition 50 (GI50) values, with markedly stronger cytotoxic effects observed in the mutant cells (Figure S10a,b, Supporting Information). Moreover, WWQ‐03‐012 caused a dose‐dependent reduction in survival in HEL cells (JAK2^V617F^‐driven), while showing little to no effect on human normal PBMCs. We also compared its inhibitory effects on mouse normal CD34^+^ cells (isolated from bone marrow) and Ba/F3‐JAK2‐V617F cells using a cell viability assay. The results showed that WWQ‐03‐012 had minimal impact on normal hematopoietic cells after 24 h of treatment (**Figure** **6**a,b). Additionally, we tested the compound in human normal bone marrow and MPN cases with WT, Calreticulin mutant (mutCALR), or JAK2‐V617F‐mutant cells (n = 3, **Table** **1**). WWQ‐03‐012 demonstrated little to no activity in MPN cases with mutant CALR or in bone marrow from healthy donors (Figure 6c). To confirm that the mechanism involves selective stabilization of mutant JAK2, we conducted pharmacogenomic studies on the effects of WWQ‐03‐012‐mediated DESI2 inhibition in both primary mutCALR‐driven MPN samples and murine MPLW515L‐driven MPN cells. Our results showed that DESI2 inhibition did not cause JAK2 degradation in mutCALR‐ or MPLW515L‐driven MPN cells, but selectively induced degradation of mutant JAK2‐V617F in JAK2^V617F^‐driven MPN cells (Figure 6d; Figure S10c, Supporting Information). These findings confirm that DESI2 selectively regulates mutant JAK2‐V617F stability. Together, these results indicate that WWQ‐03‐012 is more potent against JAK2‐mutant cells, with minimal effects on normal or CALR‐mutant cells.

**Table 1:** Primary MPN patient sample characteristics. Normal: Normal bone marrow, WT: JAK2‐WT MPN, mutCALR: Calreticulin mutant MPN, V617F: JAK2^V617F+^ MPN, MPN‐RR: Ruxolitinib‐resistant MPN patient.

Primary patient sample	Age	Cytogenetics	Mutations
Healthy donor 1 (Normal‐1)	32	46, XX [20]	JAK2‐WT
Healthy donor 2 (Normal‐2)	65	46, XY [20]	JAK2‐WT
Healthy donor 3 (Normal‐3)	66	46, XY [20]	JAK2‐WT
Patient 1 (MPN, WT)	67	46, XY [20]	JAK2‐WT
Patient 2 (MPN, mutCALR‐1)	53	46, XY [20]	mutCALR
Patient 3 (MPN, mutCALR‐2)	83	46, XY [20]	mutCALR
Patient 4 (MPN, mutCALR‐3)	65	46, XY [20]	mutCALR
Patient 5 (MPN, MPN‐RR‐1)	44	46, XX [20]	JAK2‐V617F
Patient 6 (MPN, MPN‐RR‐2)	75	46, XY [20]	JAK2‐V617F
Patient 7 (MPN, MPN‐RR‐3)	69	46, XY [20]	JAK2‐V617F
Patient 8 (MPN, MPN‐RR‐4)	81	46, XY [20]	JAK2‐V617F
Patient 9 (MPN, V617F‐1)	29	46, XY [21]	JAK2‐V617F
Patient 10 (MPN, V617F‐2)	68	46, XX [20]	JAK2‐V617F
Patient 11 (MPN, V617F‐3)	75	46, XY [20]	JAK2‐V617F

Moreover, we compared the anti‐proliferative effects of WWQ‐03‐012 and Ruxolitinib (FDA‐approved JAK1/2 inhibitor) in MPLW515L‐driven MPN cells (JAK2‐WT). After 48 or 72 h of treatment, WWQ‐03‐012 exhibited significantly lower cytotoxicity than Ruxolitinib, underscoring the mutation‐specific effect of DESI2 inhibition in JAK2^V617F^‐driven MPNs (Figure S10d, Supporting Information). In contrast, WWQ‐03‐012 showed a dose‐dependent reduction in survival in JAK2^V617F^‐driven cell lines (HEL and Ba/F3‐JAK2‐V617F), demonstrating superior efficacy compared to Ruxolitinib after 24 or 72 h of treatment (Figure S10e, Supporting Information). We then tested combination treatments of Ruxolitinib with WWQ‐03‐012 on JAK2^V617F^‐driven cells (Ba/F3‐JAK2‐V617F), and observed a significant improvement in cell killing effects, with cell viability decreasing from 60% to below 10% within 24 h (Figure S10f, Supporting Information). And we have conducted additional signaling western blot analysis following combination treatment. Our results show that while the primary effect of the combination is driven by WWQ‐03‐012′s strong inhibitory action, the combined treatment notably enhances cell‐killing efficacy (Figure S10f,g, Supporting Information). We also treated DESI2 knockdown JAK2‐V617F^+^ HEL cells with Ruxolitinib and observed a significant increase in cell killing, along with enhanced inhibition of downstream signaling pathways, compared to the scrambled control group (Figure 6e; Figure S10h, Supporting Information). These findings demonstrate that DESI2 depletion significantly sensitizes cells to Ruxolitinib, consistent with the enhanced efficacy observed with the combination of WWQ‐03‐012 and Ruxolitinib. This further supports the synergistic effect of the combination in modulating JAK2/STAT signaling pathways.

Given the potential for JAK2 kinase inhibitors to induce JAK2‐persistent cells, we evaluated the efficacy of the DESI2 inhibitor WWQ‐03‐012 in Ruxolitinib‐resistant conditions using two complementary strategies. First, induction of ruxolitinib persistence: We induced Ruxolitinib persistence (RP) by culturing HEL cells under high‐serum conditions (50%), a well‐established method for promoting resistance to various inhibitors.^[^ ^47^ ^]^ Second, Generation of Ruxolitinib‐Resistant Cell Lines: Stable Ruxolitinib‐resistant (RR) HEL or Ba/F3‐JAK2‐V617F cell lines were generated by subjecting them to dose escalation with 2 µm ruxolitinib, gradually increasing to 5 µm Ruxolitinib following 5 Gy X‐ray irradiation. These resistant clones were then treated with WWQ‐03‐012, and their responses were compared to parental cells. Both strategies demonstrated that WWQ‐03‐012 effectively overcomes Ruxolitinib resistance, significantly suppressing the proliferation of resistant cells (Figure S11a–c, Supporting Information). Additionally, we collected primary cells from Ruxolitinib‐resistant/persistent patients (n = 4, Table 1) and tested them with WWQ‐03‐012, either alone or in combination with Ruxolitinib. The relapsed patient samples in our study are defined as those that experienced loss of response after initial Ruxolitinib treatment, primary resistance (never responded to treatment), or disease progression during treatment. Notably, we observed that WWQ‐03‐012 demonstrated significant efficacy against Ruxolitinib‐resistant cells (MPN‐RR) (Figure 6f; Figure S11d, Supporting Information). This suggests its potential to overcome resistance and improve therapeutic outcomes. When used together, the combination treatment reduced the cell kill rate from 30–40% to below 10% within 24 h. These findings highlight the promising role of DESI2 inhibition in addressing Ruxolitinib resistance (Figure 6g).

We then carried out a non‐invasive implantation study with luciferase‐positive HEL cells (HEL‐Luc^+^‐GFP^+^) (n = 5/group) in female NSG mice (Figure 6h). Consistently, we observed that tumor growth was significantly slower in the WWQ‐03‐012 (12.5 mg kg^−1^, BID) treated mice compared to their littermate control (Vehicle), as measured by in vivo bioluminescence. This resulted in a statistically significant decrease in leukemia burden in the WWQ‐03‐012 treated mice compared to the vehicle control group (Figure 6h,i), showing comparable potency to Ruxolitinib (50 mg kg^−1^, QD). Additionally, aliquots of the bone marrow samples from treated mice showed an ≈4‐fold reduction in human JAK2‐V617F levels (GFP^+^JAK2^+^) compared to the vehicle group (Figure 6j). Taken together, these results suggest that the reduction in tumor burden is likely due to the effects of DESI2 inhibition, particularly through the modulating of JAK2‐V617F levels. Thus, in a therapeutic context, DESI2 robustly affects the expansion of JAK2 mutant cells in vivo. Moreover, no significant difference in body weight was observed between the WWQ‐03‐012‐treated mice and the other groups after 24 days of treatment (Figure 6k).

Collectively, these data suggest that blocking DESI2 could represent a novel clinical therapeutic strategy for JAK2 mutant leukemias.

### 2.7 WWQ‐03‐012 Alone or Combined with Ruxolitinib Shows Remarkable Efficacy in JAK2^V617F^‐Driven MPN In Vivo

Given that the effects of Ruxolitinib (and other type I JAK inhibitors) with chronic use can lead to reactivation of the JAK2 pathway through JAK2‐dependent heterodimeric complexes, enhancing pJAK2 (including JAK2‐V617F) phosphorylation and resulting in the emergence of Ruxolitinib‐resistant cells,^[^ ^10^, ^11^ ^]^ we next assessed the therapeutic effects of WWQ‐03‐012 alone or combined DESI2 and JAK2 inhibition on the progression of JAK2^V617F^‐driven MPN using JAK2‐V617F transgenic mouse. To generate this model, we cloned the full coding sequence of human JAK2‐V617F into the HS321/45‐vav vector and injected the plasmid into C57BL/6XDBA/2 F2 mouse pronuclei. Transgenic mice were identified by PCR amplification of a 594 bp region of the JAK2V617F coding sequence from genomic DNA extracted from tail samples. The use of the second JAK2‐V617F transgenic model was driven by two key considerations. First, while the previous JAK2‐V617F knock‐in model predominantly represents the PV subtype, the transduced model captures both PV and ET subtypes, thereby broadening the therapeutic relevance of our study. Additionally, the transduced model offers faster breeding rates, which were necessary to meet the high animal demands for treatment studies. Although the transduced model presents a mixed PV and ET phenotype, potentially leading to less pronounced erythroid neoplasia compared to the knock‐in model, we evaluated disease progression using RBC, white blood cell (WBC), and platelet counts, which showed significant abnormalities at baseline before treatment (data not shown).

**Figure 7:**
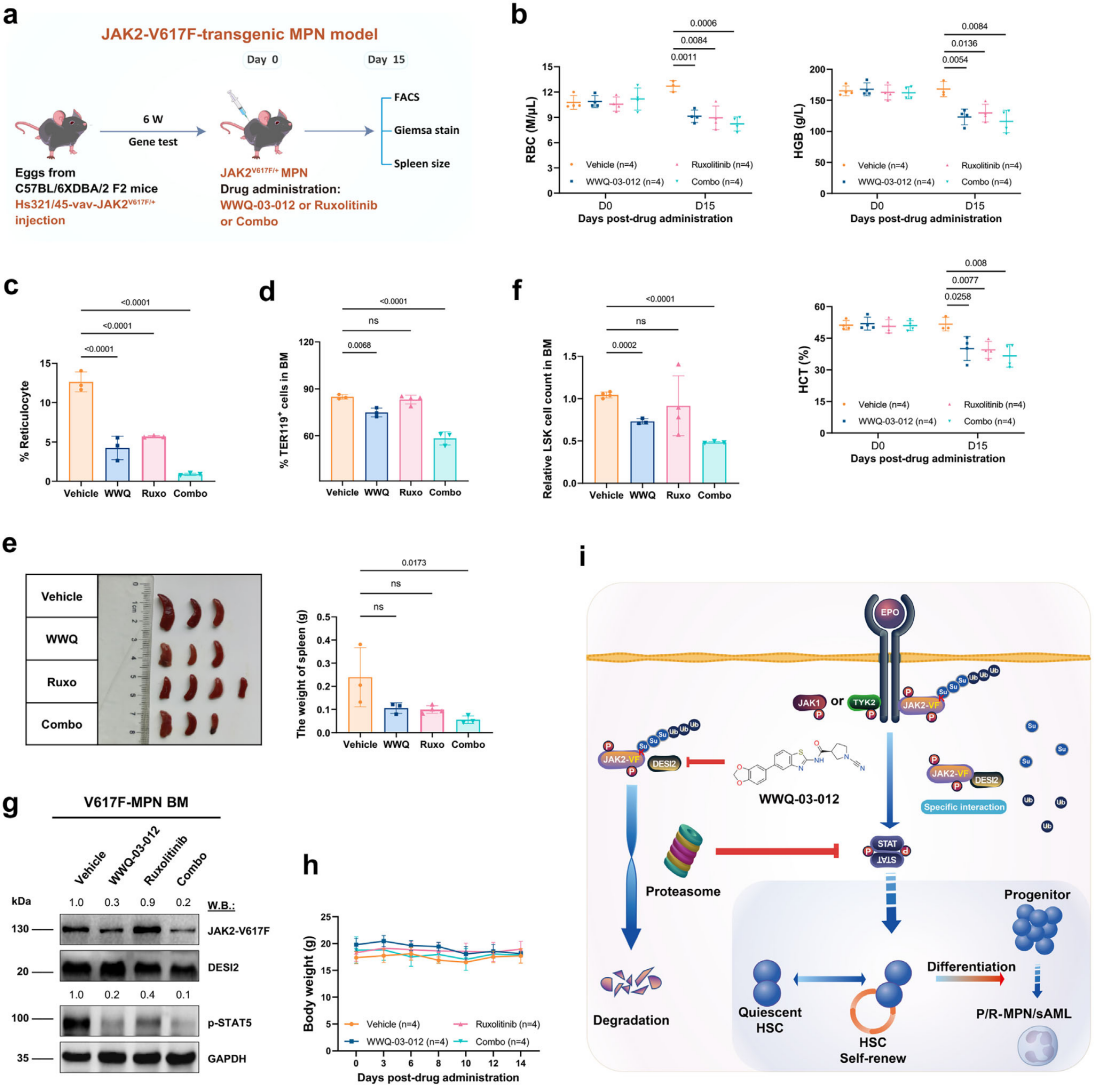
WWQ‐03‐012 alone or in combination with Ruxolitinib exhibits remarkable efficacy in JAK2^V617F^‐driven MPN in vivo. a) Schematic strategy for the effects of DESI2/JAK2 inhibition on JAK2^V617F^‐driven MPN mice. b) Statistical analysis of red blood cells (RBC), hemoglobin (HGB), and hematocrit (HCT) counts in peripheral blood from the indicated group of mice, as shown in (a) after treatment. Each dot represents one mouse (n = 4). c) The proportion of immature reticulocytes in the peripheral blood of mice was determined. d) The percentage of TER119^+^ cells detected after treatment was analyzed to assess the impact on erythropoiesis and the overall hematopoietic function in the treated mice. e) Spleen size was measured and analyzed to assess the impact of treatment on splenomegaly, a common feature of JAK2^V617F^‐driven MPN. Representative photographs of spleens are shown, with the statistical data presented on the right. f) The relative number of Lin‐ Sca‐1^+^ c‐Kit^+^ (LSK) cells in the bone marrow was assessed using flow cytometry and statistically analyzed following treatment with different conditions. g) Bone marrow samples from (a) were collected, and mononuclear cells were isolated after red blood cell lysis. Western blot analysis was then performed to measure JAK2 levels and downstream signaling proteins using specific antibodies following the indicated treatments. h) Body weights of mice treated for up to 15 days. i) Graphic abstract of this work: WWQ‐03‐012 selectively degrades mutant JAK2, leading to MPN cell death by targeting DESI2 enzymatic activity. P: phosphorylation, Su: SUMO protein, Ub: ubiquitin, HSC: Hematopoietic stem cell, P/R: Persistent/Resistant, MPN: Myeloproliferative neoplasms, sAML: secondary Acute myeloid leukemia. Data are presented as the mean ± SD. The ordinary one‐way ANOVA.

We then treated MPN mice model at 8 weeks of age with WWQ‐03‐012 (12.5 mg kg^−1^, i.p., BID), Ruxolitinib (Ruxo, 50 mg kg^−1^, i.p.) alone and in combination with 03–012 (Combo) (**Figure** **7**a). We observed that WWQ‐03‐012 treatment significantly reduced the JAK2‐V617F‐mediated erythroid burden, characterized by the reduced RBC, HGB, and HCT counts, as well as reticulocytes in peripheral blood from the indicated groups of mice (Figure 7b,c; Figure S12a, Supporting Information). In line with this, we also observed that WWQ‐03‐012 significantly reverted the erythroid hyperplasia with in the bone marrow (Figure 7d), and also ameliorated the splenomegaly in MPN mice (Figure 7e). Although Ruxolitinib also reduced RBC counts and reticulocytes in MPN mice, it had limited effect on the erythroid hyperplasia and LSCs that were largely diminished in WWQ‐03‐012‐treated MPN mice (Figure 7b–f; Figure S12b, Supporting Information). Notably, combined WWQ‐03‐012 and Ruxolitinib showed further therapeutic benefits for MPN compared to their single treatments. Similar with the findings in vitro and in other JAK2^V617F^‐driven MPN models in vivo, WWQ‐03‐012 also significantly reduced the JAK2‐V617F protein levels and downstream signal transduction in MPN mice (Figure 7g). Notably, MPN mice with WWQ‐03‐012 administration showed comparable body weights as control group, indicating minimal toxicity of this administration (Figure 7h). These results highlight the synergistic therapeutic efficacy of combination treatment in targeting JAK2^V617F^‐driven MPN, which show clinical implications for improving treatment outcomes in patients resistant or insensitive to Ruxolitinib.

To further evaluate the in vivo selectivity of WWQ‐03‐012 against JAK2^V617F/+^ cells, we administered WWQ‐03‐012 to littermate wild‐type control mouse models to assess its effect on JAK2‐WT clones. Littermate JAK2^+/+^ mice (JAK2‐WT, 6–8 weeks old, n = 6–8) were treated with either WWQ‐03‐012 or Ruxolitinib. Peripheral blood was collected for statistical analysis of WBC, RBC, and platelet counts, and bone marrow was harvested for flow cytometric analysis of various hematopoietic cell populations, including TER119⁺ (erythroid), B220⁺ (B lymphoid), CD3e⁺ (T lymphoid), Gr1⁺Mac1⁺ (myeloid), and Lin‐ Sca‐1⁺ c‐Kit⁺ (LSK) cells, on day 15 post‐treatment. Treatment with either WWQ‐03‐012 or Ruxolitinib exhibited minimal impact on peripheral blood parameters, including WBC, RBC, and platelet (Figure S13a, Supporting Information). Analysis of bone marrow (BM) from JAK2^+/+^ mice revealed no significant alterations in the proportion of TER119⁺ erythroid cells, B220⁺ B cells, CD3e⁺ T cells, and Gr1⁺Mac1⁺ myeloid cells (Figure S13b–d, Supporting Information), as well as LSK cells (Figure S13e, Supporting Information). These results suggest that DESI2 inhibition does not impair erythropoiesis or multilineage hematopoietic differentiation in wild‐type settings. Furthermore, we tested the combination of Ruxolitinib and WWQ‐03‐012 in healthy mice from the same cohort. The combination treatment did not significantly alter key hematological parameters, such as RBC, WBC, or platelet counts, indicating no noticeable toxicity (data not shown). These results support the potential clinical use of the combination without significant adverse effects.

Together, these results further support the therapeutic potential of DESI2 inhibition in JAK2^V617F^‐driven leukemias (Figure 7j), demonstrating its ability to target leukemic cells while sparing wild‐type hematopoiesis and preserving multilineage differentiation. Moreover, these findings have important clinical implications, particularly for patients resistant or insensitive to standard therapies.

## 3 Discussion and Conclusion

Mutations in the Janus Kinase 2 (JAK2) gene, which result in constitutive kinase activation, represent the most common genetic event in myeloproliferative neoplasms (MPN).^[^ ^48^, ^49^, ^50^ ^]^ MPNs are a group of clonal disorders of hematopoietic stem cells that include PV, ET, and PMF. A subset of MPN patients transforms to secondary acute myeloid leukemia (sAML), a condition that is resistant to traditional chemotherapy, with limited treatment options and a poor prognosis, resulting in a 5‐year survival rate of ≈12%.^[^ ^6^, ^7^, ^8^, ^9^ ^]^ JAK2 kinase inhibitors, such as Ruxolitinib and Baricitinib (both JAK1/JAK2 inhibitors),^[^ ^51^, ^52^, ^53^ ^]^ provide clinical benefits for MPN patients. However, their therapeutic potential is limited by the inhibition of WT JAK2, which caused toxicity to normal cells. Additionally, small molecule inhibitors targeting mutant JAK2 kinase activity often lead to the emergence of drug resistance.^[^ ^10^, ^11^, ^54^ ^]^ Therefore, a novel strategy that targets mutant JAK2 for degradation by harnessing the cell's intracellular degradation machinery would offer a more effective and clinically advantageous approach for MPN and sAML patients.

Here, using a combination of mass spectrometry‐based proteomics and genetic studies, we identified the deSUMOylases DESI2 as a novel component of the JAK2‐V617F complex that regulates its stability. Importantly, through an in‐house compound library screening, followed by chemical proteomics and structure optimization, we discovered a first‐in class inhibitor, WWQ‐03‐012, which selectively degrades mutant JAK2 by targeting DESI2 enzymatic activity both in vitro and in vivo (IC_50_ of 47.3 nm, DC_50_ of 543.6 nm in HEL). DESI2 is a protein containing a conserved DUF862 domain and belongs to the cysteine isopeptidase family. Previous studies have demonstrated that DESI2 possesses deubiquitinating activity and isopeptidase activity against ubiquitin conjugated through Lys48 and Lys63.^[^ ^18^ ^]^ However, its broader functional roles remain underexplored. The first‐in‐class inhibitors that we discovered and optimized may serve as useful tools for probing DESI2‐mediated signaling and for identifying coactivators and regulatory partners, such as the PI3K/AKT/mTOR pathway.^[^ ^19^, ^55^ ^]^ Moreover, we have validated DESI2 as a new therapeutic target and identified a novel therapeutic strategy for JAK2‐V617F‐positive leukemia. Unlike existing small‐molecule kinase inhibitors that target JAK2, this approach efficiently degrades mutated JAK2, thereby eliminating cancer cells and overcoming drug resistance.

To further investigate the functional landscape of DESI2 inhibition, we performed proteomic analyses following treatment with WWQ‐03‐012 or DESI2 knockdown. These analyses confirmed JAK2‐V617F as a key protein, ranked in the top 5% (11/273) of those significantly regulated by DESI2, supporting the main conclusions of our study (Figure S9d, Supporting Information). Notably, neither DESI2 KD nor inhibition reduced the protein levels of other JAK family members, highlighting the specificity of DESI2 for JAK2 (Figure 3e,f; Figure S9e, Supporting Information). Additionally, our proteomic analysis revealed that WWQ‐03‐012 affected several signaling proteins involved in tumorigenesis, such as DNMT1 (DNA Methyltransferase 1), HELLS (Helicase) and BLM (Bloom Syndrome RecQ‐Like Helicase), suggesting their potential roles in DESI2‐mediated processes (Figure S9d, Supporting Information). Further investigation into their involvement in DESI2‐mediated processes is currently ongoing, and we are gradually validating these potential targets as part of our future research focus.

Previously, we successfully identified USP10 inhibitors selectively targeting FLT3‐ITD, JOSD1 inhibitors selectively targeting JAK2, and USP47 inhibitors targeting mutant EZH2 in AML, MPN, or DLBCL.^[^ ^25^, ^28^, ^31^ ^]^ These findings demonstrate that this novel approach has been effective and warrant further investigation as an alternative treatment strategy for a broader range of diseases. Recently, five DUB inhibitors have been under clinical studies, including a promising candidate, the USP1 inhibitor KSQ‐4279, which is currently in a Phase 1 trail to evaluate its safety and clinical activity, both as a monotherapy and in combination therapies, in patients with advanced solid tumors (data from NCI). These developments, together with our findings, suggest that targeted therapies designed to selectively degrade constitutively activated oncogenic proteins can overcomes drug resistance and provided a more effective treatment strategy. Unlike traditional kinase inhibitors, these therapies simultaneously inhibit both the enzymatic and scaffolding functions of oncogenic proteins, offering potential advantages in treating “druggable” and even “undruggable” targets. This approach may prove more efficacious and durable, addressing a significant unmet need in cancer therapy.

Due to the lack of clinical databases linking JAK2 mutations to AML prognosis, we turned from in‐silico analysis to Western blot analysis of our clinical samples, focusing on the post‐translational regulation of JAK2‐V617F by DESI2. Our analysis of JAK2 wild‐type and mutant leukemia cell lines, along with primary samples from normal bone marrow, JAK2‐WT and JAK2‐V617F‐positive MPN patients (n = 3/group), revealed significantly higher protein levels of both DESI2 and JAK2‐V617F in the mutant cells (Figure 3a–d). This suggests that DESI2 may act as a potential clinical biomarker for JAK2‐V617F mutant leukemia. Furthermore, we found that inhibition of DESI2 selectively led to the degradation of JAK2‐V617F and inhibited growth in primary patient samples (Figure 6; Figure S10, Supporting Information). These results strongly support our hypothesis that DESI2 is crucial for maintaining the stability of JAK2‐V617F, positioning it as a promising therapeutic target for JAK2^V617F^‐driven malignancies.

To further establish the mutant‐specificity of DESI2, we utilized orthogonal methods and conducted additional experiments, including mass spectrometry (Figure 1a; Figure S3c,d, Supporting Information), co‐IP assays (Figure 1d), immunofluorescence (IF) analyses (Figure 1e,f), DESI2 rescue assays in KD cells (Figure S2c–e, Supporting Information), and AlphaFold3 modeling to predict the binding modes of DESI2 with both WT and JAK2‐V617F. Compared to JAK2‐WT, the binding pattern of DESI2 to JAK2‐V617F involves interactions with two distinct regions and the formation of three additional hydrogen bonds, indicating enhanced binding affinity and structural stabilization. These studies uncovered the mechanistic basis for DESI2's mutant selectivity (Figure S4, Supporting Information). Building upon these structural and biochemical findings, we next sought to confirm that the DESI2–JAK2 interaction is indeed mutation‐dependent rather than an artifact of different cellular backgrounds. Our findings provide clear mechanistic evidence that the DESI2–JAK2 interaction is specifically driven by the V617F mutation rather than reflecting background‐dependent variability between different cell lines. While our initial proteomic identification employed distinct human leukemia cell lines (HEL and K562) to ensure biological relevance, the subsequent validation using isogenic and single‐background systems rigorously confirmed the mutation‐dependent nature of this interaction. The combination of Ba/F3 isogenic lines, and controlled expression in the same K562 background offers a robust experimental framework that minimizes the confounding influence of genomic heterogeneity (Figure S1b,c, Supporting Information). Together, these data strengthen the mechanistic conclusion that DESI2 selectively stabilizes the JAK2‐V617F mutant through direct interaction, underscoring its potential as a mutation‐specific therapeutic target in MPN and post‐MPN AML. Mechanistically, our findings demonstrate that DESI2 stabilizes mutant JAK2 by mediating its deubiquitination and deSUMOylation, a mechanism not observed with wild‐type JAK2. Notably, in addition to our pharmacogenomic analyses of DESI2 inhibition in mutCALR and MPL515L‐driven MPN primary cells, we conducted in vivo studies to further validate this effect. These included CDX studies with DESI2 knockout in JAK2 wild‐type lines (K562‐Luc) (Figure S7, Supporting Information) and a littermate wild‐type JAK2^+/+^ control mouse model treated with DESI2 inhibition (Figure S13, Supporting Information). Importantly, the results from these experiments, including the assessment of the mutant cell population under WWQ‐03‐012 treatment in CDX models and transgenic mice, compared to wild‐type controls, consistently demonstrated the mutant‐specificity of DESI2 both in vitro and in vivo. Collectively, these findings strongly support the hypothesis that DESI2's regulatory role is confined to the mutant form of JAK2, establishing it as a promising and mutation‐selective therapeutic target for JAK2‐V617F–driven malignancies.

In addition, the homozygous JAK2‐V617F cell lines, which we have primarily used in our studies, may offer a more suitable model for investigating JAK2‐V617F‐DESI2 interactions. However, we acknowledge that the heterozygous JAK2‐V617F SET2 cell lines, which retain a wild‐type copy of JAK2, could introduce additional complexity in studying these interactions. The potential interference of wild‐type JAK2 with the binding of DESI2 to the mutant protein may impact the results. While we have not yet investigated SET2 cells, we agree that including them could offer valuable insight into the role of DESI2 in MPN and post‐MPN AML. In the DepMap database, HEL does not show a dependency on DESI2 for normal growth, which contrasts with our findings. While the DepMap provides broad, population‐level data, our study focuses on targeted, cell‐specific experimental conditions. In our experiments, we observed that DESI2 depletion significantly impacts JAK2‐V617F stability, signaling, and cell viability, particularly in JAK2‐V617F‐dependent cells. This discrepancy may arise from differences in experimental settings or the specific context of the cell lines used. Our results strongly support a critical role for DESI2 in maintaining the viability of JAK2^V617F^‐driven cells.

Moreover, DESI2 stabilizes JAK2‐V617F through deSUMOylation and deubiquitylation of its protein at K962. The interplay between deSUMOylation and deubiquitination in regulating JAK2‐V617F stability is a critical aspect of our study. Our results suggest that DESI2 modulates both post‐translational modifications to stabilize JAK2‐V617F (Figures 2e–i and 5i). Mass spectrometry identified K962 as a key residue modified by both SUMOylation and ubiquitination, highlighting the dual role of DESI2 in regulating these modifications (Figure 2j,k; Figure S3, Supporting Information). Furthermore, inhibition of SUMOylation by ML‐792 reduced both SUMOylation and ubiquitination of JAK2‐V617F, and DESI2 knockdown compromised this response (Figure 2h), indicating that DESI2's regulation of JAK2 stability is closely linked to SUMOylation followed by ubiquitination. Proteasomal inhibition with MG132 rescued JAK2‐V617F degradation in DESI2‐depleted cells (Figure 2d; Figure S2g, Supporting Information), suggesting that SUMOylation precedes ubiquitination in this regulatory pathway. These findings underscore the cooperative role of deSUMOylation and deubiquitination in maintaining JAK2‐V617F stability and provide a mechanistic framework for understanding how DESI2 governs mutant JAK2 in myeloproliferative neoplasms.

Building on this, the genetic depletion of DESI2 significantly suppresses both JAK2 mutant cells growth and MPN disease onset in vitro and in vivo. Our studies support the notion that targeting DESI2 could induce the degradation of mutant JAK2 as an alternative to JAK2 kinase inhibition, offering a potential solution to overcome drug resistance while minimizing the adverse side effects observed in current clinical treatments, such as impacts on wild‐type hematopoiesis. Specifically, our findings in various preclinical models highlight the therapeutic potential of DESI2 inhibition (either genetically or pharmacologically) in JAK2^V617F^‐driven MPN, both as a monotherapy or in combination with Ruxolitinib, particularly for patients resistant or insensitive to Ruxolitinib (Figures 4, 6, and 7; Figures S6, S7, and S10–S13, Supporting Information). These findings not only underscore the critical role of DESI2 inhibition in addressing Ruxolitinib resistance but also introduce a novel therapeutic strategy targeting mutated JAK2 signaling in MPN and sAML. Further translational research is essential to explore the clinical applications of this approach, which could lead to encouraging outcomes in future trials. This strategy represents a promising avenue for developing more effective treatments for patients with JAK2^V617F^‐driven diseases.

WWQ‐03‐012 and XL‐106C were both derived from SB1‐F‐70 but demonstrate subtype selectivity for DESI2 (IC_50_ of 47.3 nm) and JOSD1(IC_50_ > 10 000 nm), suggesting that structural optimization can enhance selectivity for new protein targets beyond those of their parent ligands. Both JOSD1 and DESI2 have been validated as stabilizers of mutant JAK2, with XL‐106C shown to bind DESI2 (Figure 5a), although it is less potent against DESI2 enzymatic activity (IC_50_ > 10 000 nm). Therefore, combining a JOSD1 inhibitor with a DESI2 inhibitor could be more potent than monotherapy by simultaneously targeting multiple pathways involved in mutant JAK2 stabilization and resistance, thus representing a promising new therapeutic direction.

## 4 Experimental Section

### Cell Lines and Cell Culture

All cell lines used in this study were purchased from the American Type Culture Collection (ATCC) (Manassas, VA, USA). K562‐luc^+^ cells were obtained from Shanghai Model Organisms Center, Inc. Detailed information on cell lines and culturing conditions, including the Research Resource Identifiers (RRIDs) for each cell line, is provided in the Supporting Information.

### Biologic Reagents

Detailed information on biological reagents is provided in the Supporting Information.

### Chemical Compounds

SB1‐F‐70, XL‐106C, and WWQ‐03‐012, were designed and synthesized in‐house (synthesis described in supplemental methods). Results of UPLC‐MS analysis was consistent with reported purity and molecular weight. Ruxolitinib was purchased from Targetmol. Inhibitors were dissolved in DMSO to obtain 10 mm stock solutions. Serial dilutions were then made, to obtain final dilutions for cellular assays with a final concentration of DMSO between 0.2 and 0.5%. Cycloheximide (CHX) and MG132 were purchased from Sigma–Aldrich.

### Cell Transfections

Details of cell transfections are provided in the Supporting Information.

### Immunoblotting and Immunoprecipitation

Protein lysate preparation, immunoblotting, and immunoprecipitation were carried out as previously described.^[^ ^23^ ^]^

### Proliferation and Drug Combination Studies

Proliferation and drug combination studies were carried out as previously described.^[^ ^26^ ^]^

### shRNA Knockdown and CRISPR Knockout assay

Details of the shRNA knockdown and CRISPR knockout assays are provided in the Supporting Information.

### Immunofluorescence (IF) and Confocal Microscopy

Detailed information on IF and confocal microscopy is provided in the Supporting Information.

### Mass Spectrometry‐Based Proteomics

Detailed information on the proteomic analysis of JAK2 complexes via co‐immunoprecipitation and mass spectrometry is provided in the Supporting Information.

For compounds high‐throughput target mass analysis, combined with competitive activity‐based protein profiling with quantitative mass spectrometry, using biotin‐labeled small molecule compounds to compete with unlabeled small molecules. Biotin‐streptavidin pull‐down was performed and the binding effects of small molecules on different proteins were quantitatively measured ability to block binding of proteins by proteomics in HEK‐293T lysates. Details were performed as previously described.^[^ ^45^ ^]^ The raw mass spectrometry data generated has been deposited in the PRIDE public repository under accession code: PXD065727. Token: ezZNjoG3fUi6. The raw quantitative proteomic profiling data generated has been deposited in the PRIDE public repository under accession code: PXD065708. Token: guKbxIfQc0u8.

### Protein Expression and Purification

The JOSD1 protein expression and purification were carried out as previously described.^[^ ^25^ ^]^ A construct of human DESI2 covering residues 1–194 aa in the pET28a vector was obtained from PPL and over‐expressed in E. coli BL21 (DE3) in TB medium in the presence of 50 mg mL^−1^ of kanamycin. The cells were grown at 37 °C to an OD of 0.8, cooled and induced, then collected by centrifugation. Cell pellets were sonicated, and the resulting lysate was centrifuged at 30 000 g for 30 min. Lysate supernatant was mixed with Ni‐NTA beads (Qiagen), transferred to an FPLC‐compatible column and the bound protein was washed with buffers, eluted and concentrated followed the previously description. Recombinant human DESI2 protein was concentrated to 20 mg mL^−1^, and aliquoted for long‐term storage at −80 °C.

### Target‐Engagement Assay

Using biotin‐labeled small molecule compounds to compete with unlabeled small molecules for target binding experiments. Details were performed as previously described.^[^ ^30^ ^]^ Briefly, to verify the binding effect of small molecules and DESI2. Human AML cells driven by the JAK2‐V617F mutation (HEL cell lysate), 2 mg protein per sample, first pretreated with Biotin‐F‐70 (50 µm) for 4 h, then add SB1‐F‐70 (0, 5, 25, 100 µm) and incubate for 4 h. Biotin‐streptavidin pull‐down was performed and the binding effects of small molecules on DESI2 were visualized by western blotting. With XL‐177A (specific USP7 inhibitor) as a negative control.

### Ubiquitin‐AMC/Rho Assay

Details were performed as previously described.^[^ ^25^ ^]^ DESI2 activity assay. Briefly, recombinant DESI2, residues, was tested for its activity in a Ubiquitin‐AMC/Rho assay in presence or absence of inhibitors. For this assay, 10 nM DESI2 were pre‐incubated with different concentrations of inhibitors or DMSO as a control in 50 mm HEPES pH7.6, 0.5 mm EDTA, 11 µm ovalbumin, 5 mm DTT. The reaction was incubated for 6 h at room temperature prior to the addition of 1 µm Ubiquitin‐AMC/Rho 110: R&D #U‐550/U‐555 (provided by Prof. Yiming Li) substrate. The initial rate of the reaction was measured by collecting fluorescence data at one‐minute interval over 30‐min period using a Clariostar fluorescence plate reader at excitation and emission wavelength of 345 and 445 nm for Ub‐AMC, 485 and 535 nm for Ub‐Rho, respectively. The calculated initial rate values were normalized to DMSO and plotted against inhibitor concentrations to determine IC50 values.

### MPN Patient Cells

Mononuclear cells were isolated from WT JAK2, mutCALR^+^ or JAK2‐V617F^+^ MPN patient samples. Cells were tested in liquid culture (RPMI 1640 supplemented with 10% FBS) in the presence of drug. All blood and bone marrow samples from MPN patients, or PBMC and bone marrow samples from healthy donors were obtained under the approval of the Tongji Hospital Review Board (TJ2023‐007). The use of these clinical samples was conducted in strict accordance with the Declaration of Helsinki and was first approved by the Ethics Committee of Tongji Hospital, Tongji University, in March 2022 (Approval Number: K‐2022‐004). Written informed consent was obtained from all participants involved in the study. The patient's information was list on Table 1.

### Animal Study

Murine tumor xenograft models were performed as previously described.^[^ ^31^ ^]^

For the tail vein non‐invasive in vivo bioluminescence study, bioluminescence imaging was performed as described previously.^[^ ^28^ ^]^ Briefly, for administration to female NSG mice (6–8 weeks of age; Shanghai Model Organisms Center, Inc.), virus‐ and mycoplasma‐free HEL‐Luc^+^ cells (Mock, SCR, 72609 or 67575) or K562‐Luc^+^ cells (SCR, KO1 or KO2) were washed and resuspended in 1× PBS and administered via IV tail vein injection (3×10^6^ or 1×10^6^ cells/250 µL). A sample size of no fewer than six mice per treatment group was chosen to ensure statistical significance. Anesthetized mice were imaged every three days following IV‐injection of Luc^+^ cells to generate a baseline, which was used to establish treatment cohorts with matched tumor burden (mice were randomized and investigators were blinded to group allocation). Total body luminescence was measured as previously described.^[^ ^28^ ^]^ The percentage of Luc (GFP) or JAK2 levels as measured by flow cytometry using both GFP and a JAK2 antibody in bone marrow samples from different transplanted mice (n = 6). The survival curve of transplanted NSG mice was generated using the Kaplan–Meier method.

For WWQ‐03‐012 treatment, virus‐ and mycoplasma‐free HEL‐Luc^+^ cells were washed and resuspended in 1× PBS and administered via IV tail vein injection (3×10^6^ cells/250 µL). Drug treatment commenced two days after cell injection. Mice were treated with vehicle (5% DMSO in HKI Solution (0.5%Methocel/0.4%Tween80)), i.p. BID) (n = 5), WWQ‐03‐012 (12.5 mg kg^−1^ in HKI Solution, i.p. BID) (n = 5), Ruxolitinib (50 mg kg^−1^ in HKI Solution, i.p. QD) (n = 4) for the indicated times. Correlation between luciferase‐positive leukemia burden as measured by Bright Glo assay and Luminoskan every 3 days, and bioluminescent images of representative mice with matched starting leukemia burden were taken. The body weights of transplanted mice were measured every two days for up to 24 days.

### JAK2^V617F^‐Driven MPN Mice Model

JAK2^V617F^‐knockin mice were generated as previously described.^[^ ^39^ ^]^ The JAK2^V617F^ mouse model for therapeutic study was generously provided by Dr. Lei Zhang from the State Key Laboratory of Experimental Hematology, National Clinical Research Center for Blood Diseases, Tianjin, China. Both models are heterozygous for JAK2‐V617F, but they differ in terms of their characterization and specific phenotypic features. Briefly, they utilized transgenic mice expressing the mutated enzyme specifically in the hematopoietic system driven by a vav gene promoter. The full coding sequence of human JAK2^V617F^, along with its 3′ noncoding region, was cloned into the HS321/45‐vav vector. The plasmid DNA was then digested with SacII to remove the pBSIISK backbone and subsequently used for injection into the pronuclei of eggs from C57BL/6XDBA/2 F2 mice. Transgenic mice were identified through PCR amplification of genomic DNA extracted from tail samples, using primers TACAACCTCAGTGGGACAAAGAAGAAC and CCATGCCAACTGTTTAGCAACTTCA, which cover a 594 bp region of the JAK2^V617F^ coding sequence. Both JAK2^V617F+^ and their littermate wild‐type control mice were treated with WWQ‐03‐012 (12.5 mg kg^−1^ in HKI Solution, i.p. BID) or Ruxolitinib (50 mg kg^−1^ in HKI Solution, i.p. QD). On day 15 post‐treatment, complete blood counts were performed, and 70 µL of peripheral blood from each mouse was analyzed using a Mindray complete blood count analyzer. Bone marrow was also harvested for flow cytometric analysis.

The transplanted JAK2^V617F+^ mouse model was generated by Dr. Baobing Zhao from the Key Lab of Chemical Biology, School of Pharmaceutical Sciences, Cheeloo College of Medicine, Shandong University, Jinan, Shandong, China.^[^ ^44^ ^]^ Briefly, for DESI2 silencing assays, bone marrow cells were purified from JAK2^V617F^‐knockin mice (CD45.2‐positive, 6–8 weeks old) using a c‐kit^+^ selection kit (Stem Cell Technologies, catalog 18757), and cultured in expansion medium overnight. shDESI2‐viral transduction was performed twice on the second day, with the knockdown virus being EGFP‐tagged. EGFP‐positive cells (DESI2 KD cells) were sorted 48 h post‐infection. A total of 2 × 10^5^ cells were transplanted into lethally irradiated (10 Gy) recipient mice (CD45.1‐positive, B6‐LY‐5.2/Cr). Recipient mice were then kept on antibiotic water (1.1 mg mL^−1^ neomycin and 2000 U mL^−1^ polymyxin B [both Millipore Sigma]) for 2 weeks, followed by regular water. Four weeks post‐transplantation, bone marrow was collected for qPCR and Western blot analysis. For the analyses of complete blood counts, peripheral blood (70 µL from each mouse) was collected from the retro‐orbital vein in EDTA‐coated tubes and analyzed by a Mindray complete blood count.

Mice were sacrificed according to the experimental protocols or when they met prespecified endpoints defined by the IACUC. Animal technicians were blinded to expected outcomes. All animal studies were performed in specific‐pathogen‐free, Helicobacter‐free facilities at Tongji University, Shandong University and the Chinese academy of Sciences Animal Resource Center following national, state and institutional guidelines, including the ARRIVE guidelines, and according to protocols approved by the Institutional Animal Care and Use Committee of Tongji University, Shandong University and the Chinese academy of Sciences (2022‐PD‐137, 2023‐DW‐SB‐046).

### Homing Assay

c‐Kit^+^ cells purified from JAK2‐V617F mouse bone marrow, were infected with retroviruses encoding shDESI2 for two rounds. LSK^+^GFP^+^ cells were quantified and transplanted into lethally irradiated recipients. 18 h post‐transplantation, bone marrow cells from the recipients were collected, and the number of LSK^+^GFP^+^ cells was measured. The homing rate was calculated as the ratio of LSK^+^GFP^+^ cells in the recipient mice to the total transplanted ones.

### Statistical Analyses

Data are presented as mean ± SD or SEM as indicated. Student's t‐test, ordinary one‐way ANOVA and log‐rank test was used to determine statistically significant differences (ns: *p* > 0.05) using GraphPad Prism software.

## Conflict of Interest

The authors declare no conflict of interest.

## Supporting information



Supporting Information

## Data Availability

The data that support the findings of this study are available in the supplementary material of this article.
