# Supplementary material for: Targeting DESI2 as a Novel Therapeutic Strategy for JAK2‐Mutant Leukemias
Source: Adv Sci (Weinh). 2025 Dec 3;13(7):e15127. doi: 10.1002/advs.202515127 (PMC12866797; doi:10.1002/advs.202515127)
Supplement: Supplementary file 1 — Supporting Information [file ADVS-13-e15127-s001.docx]

**Targeting DESI2 as a Novel Therapeutic Strategy for JAK2-Mutant Leukemias**

Husheng Mei^1^*, Wuqiang Wen^2^*, Wenjun Zhang^1^*, Shujing Zhang^3^*, Ting Sun^4^*, Guiming Li^1^, Jing Zhang^4^, Shuang Qi^5^, Jie Zhou^1^, Bing Li^1^, Yunshuo Zhao^1^, Xiaotong Chen^1^, Bowen Li^1^, Yiying Xue^1^, Wang Lu^1^, Yanli Sun^6^, Jingyao Wang^7^, Hengyue Shan^7^, Shengzhe Zhang^8^, Yushan Huang^1^, Yisa Chen^1^, Wenchao Wang^5^, Qingsong Liu^5^, Wenchao Lu^6^, Li Tan^7^, Yi Ding^1^, Jianfei Fu^1^, Jun Long^1^, Lei Zhang^4^, Baobing Zhao^3^**^#^**, Aibin Liang^1^**^#^**, Baishan Jiang^2^**^#^**, Jing Yang^1^**^#^**

^1^Department of Hematology, Tongji Hospital, Frontier Science Center for Stem Cell Research, Shanghai Key Laboratory of Signaling and Disease Research, School of Life Sciences and Technology, Tongji University, Shanghai, 200092, China

^2^Department of Radiation and Medical Oncology, Medical Research Institute, Frontier Science Center of Immunology and Metabolism, Hubei Key Laboratory of Tumor Biological Behavior, Zhongnan Hospital of Wuhan University, Wuhan University, Wuhan 430071, China

^3^Key Lab of Chemical Biology, School of Pharmaceutical Sciences, Cheeloo College of Medicine, Shandong University, 250012, Jinan, Shandong, China

^4^State Key Laboratory of Experimental Hematology, National Clinical Research Center for Blood Diseases, Haihe Laboratory of Cell Ecosystem, Tianjin & CAMS Key Laboratory of Gene Therapy for Blood Diseases, Institute of Hematology & Blood Diseases Hospital, Chinese Academy of Medical Sciences & Peking Union Medical College, Tianjin 300020, China

^5^Anhui Province Key Laboratory of Medical Physics and Technology, Institute of Health and Medical Technology, Hefei Institutes of Physical Science, Chinese Academy of Sciences, Hefei, 230031, PR China

^6^Lingang Laboratory, Shanghai 200031, China

^7^Interdisciplinary Research Center on Biology and Chemistry, Shanghai Institute of Organic Chemistry, Chinese Academy of Sciences, Shanghai 201210, China

^8^Institute of Translational Medicine, Medical College, Yangzhou University, Yangzhou, 225001, PR China

*These authors contributed equally to this study.

**^#^**Correspondence:

Baobing Zhao ([baobingzh@sdu.edu.cn](mailto:baobingzh@sdu.edu.cn)),

Aibin Liang ([lab7182@tongji.edu.cn](mailto:lab7182@tongji.edu.cn)),

Baishan Jiang ([baishan_jiang@whu.edu.cn](mailto:baishan_jiang@whu.edu.cn)),

Jing Yang ([jingy@tongji.edu.cn](mailto:jingy@tongji.edu.cn)).

The authors declare no conflict of interest.

**Keywords:** DESI2, JAK2-V617F Mutation, Degrader, MPN, Drug Resistance

Contents

[Supplemental Methods 3](#_Toc205413091)

[Chemical compounds synthesis 3](#_Toc205413092)

[Cell lines and cell culture 5](#_Toc205413093)

[Biologic reagents 5](#_Toc205413094)

[shRNA Knockdown and CRISPR Knockout assay 6](#_Toc205413095)

[Immunofluorescence (IF) and confocal Microscopy 6](#_Toc205413096)

[Cell transfections 7](#_Toc205413097)

[Mass spectrometry-based proteomics 7](#_Toc205413098)

[Supplementary Figures 8](#_Toc205413099)

# **Supplemental Methods**

## **Chemical compounds synthesis**

**Scheme 1. Preparation of Compounds 106C and WWQ-03-012^a^.**

^a^Reagents and conditions: a) (R)-1-(tert-butoxycarbonyl)pyrrolidine-3-carboxylic acid, HATU, TEA, DMF, rt; b) 3,5-dimethyl-4-(4,4,5,5-tetramethyl-1,3,2-dioxaborolan-2-yl)isoxazole, Na_2_CO_3_, Pd(dtbpf)Cl_2_, dioxane/H_2_O, N_2_, 95^o^C; c) TFA, DCM, rt; d) cyanogen bromide, K_2_CO_3_, DCM/DMSO, rt

Example 1: Synthesis of (S)-1-cyano-N-(7-(3,5-dimethylisoxazol-4-yl)benzo[d]thiazol-2-yl)pyrrolidine-3-carboxamide (**WWQ-04-072**).

Step1: 5-bromobenzo[d]thiazol-2-amine (**A**, 0.86 g, 3.75 mmol), (R)-1-(tert- butoxycarbonyl)pyrrolidine-3-carboxylic acid (0.96 g, 4.5 mmol), Et_3_N (2.58 mL, 18.75 mmol) and HATU (2.15 g, 5.63 mmol) were added sequentially to anhydrous DMF (20 mL). The reaction mixture was stirred at room temperature overnight. The resulting mixture was then diluted with EtOAc (50 mL), and washed with brine (30 mL×2) to remove excess DMF. The organic layer was dried over anhydrous sodium sulfate (Na_2_SO_4_), filtered, and concentrated under reduced pressure. The crude material was then purified by flash column chromatography (PE/EtOAc) to afford tert-butyl (*S*)-3-((7-bromobenzo[d]thiazol-2-yl)carbamoyl)pyrrolidine-1-carboxylate (**B**, 1.52 g, yield 95%).

Step2: tert-butyl (*S*)-3-((7-bromobenzo[d]thiazol-2-yl)carbamoyl)pyrrolidine-1-carboxylate from step 1 (1.52 g, 3.57 mmol) was dissolved in 1,4-dioxane and H_2_O (10 mL, 9:1) before the addition of 3,5-dimethyl-4-(4,4,5,5-tetramethyl-1,3,2-dioxaborolan-2-yl)isoxazole (0.073 g, 0.45 mmol), sodium carbonate (816 mg, 0.45 mmol), and Pd(dtbpf)Cl_2_ (848 mg, 0.03 mmol). The resulting mixture was degassed by bubbling through N_2_ for 10 min, and then was heated and stirred at 95°C for 2-8 h. The reaction was then allowed to cool to room temperature, diluted with EtOAc (50 mL), and washed with saturated ammonium chloride (30 mLx2). The aqueous layer was then extracted with EtOAc (50 mL). The combined organic layers were washed with brine, dried over anhydrous sodium sulfate (Na_2_SO_4_), filtered, and concentrated under reduced pressure to afford crude material, which was then purified by flash chromatography (PE/EtOAc) to afford tert-butyl (*S*)-3-((7-(3,5-dimethylisoxazol-4-yl)benzo[d]thiazol-2-yl)carbamoyl)pyrrolidine-1-carboxylate (**C**, 1g, yield 65%).

Step3: tert-butyl (*S*)-3-((7-(3,5-dimethylisoxazol-4-yl)benzo[d]thiazol-2-yl)carbamoyl)pyrrolidine-1-carboxylate from last step was dissolved in DCM (5 mL) and treated with TFA (1 mL). The mixture was stirred at room temperature until the reaction reached completion, which was monitored by LC-MS. The crude mixture was concentrated under reduced pressure and was used in next step without further purification.

Step4: (S)-N-(7-(3,5-dimethylisoxazol-4-yl)benzo[d]thiazol-2-yl)pyrrolidine-3-carboxamide from the last step (0.77g, 2.26 mmol) was dissolved in a mixture of DCM/DMSO (10 mL) and K_2_CO_3_ (2g, 11.3 mmol) at room temperature before the addition of cyanogen bromide (310 mg, 2.26 mmol). The reaction mixture was then stirred at room temperature for 5 min, and the resulting crude product was directly purified by flash chromatography (DCM/MeOH) to afford compound **WWQ-04-072(106C)**. ^1^H NMR (400 MHz, DMSO-d_6_) δ 12.70 (s, 1H), 7.83 (d, J = 7.7 Hz, 1H), 7.56 (t, J = 7.8 Hz, 1H), 7.31 – 7.26 (m, 1H), 3.64 (dd, J = 9.6, 7.8 Hz, 1H), 3.56 (dd, J = 9.6, 5.8 Hz, 1H), 3.47 – 3.41 (m, 2H), 3.41 – 3.37 (m, 1H), 2.31 (s, 3H), 2.22 (td, J = 13.6, 7.1 Hz, 1H), 2.12 (d, J = 5.2 Hz, 3H), 2.07 (dd, J = 13.3, 6.5 Hz, 1H). ^13^C NMR (101 MHz, DMSO-d_6_) 172.25, 166.32, 158.79, 158.04, 149.19, 132.90, 127.15, 125.81, 123.96, 120.94, 117.53, 115.54, 52.45, 50.31, 43.76, 29.56, 12.02, 10.71.

(S)-N-(5-(benzo[d][1,3]dioxol-5-yl)benzo[d]thiazol-2-yl)-1-cyanopyrrolidine-3-carboxamide (**WWQ-03-012**).

^1^H NMR (400 MHz, DMSO-d_6_) δ 12.63 (s, 1H), 8.02 (d, J = 8.3 Hz, 1H), 7.94 (d, J = 1.3 Hz, 1H), 7.56 (dd, J = 8.3, 1.6 Hz, 1H), 7.35 (d, J = 1.6 Hz, 1H), 7.23 (dd, J = 8.1, 1.7 Hz, 1H), 7.02 (d, J = 8.1 Hz, 1H), 6.08 (s, 2H), 3.69 – 3.62 (m, 1H), 3.59 (dd, J = 9.6, 6.1 Hz, 1H), 3.48 (d, J = 7.7 Hz, 1H), 3.46 – 3.43 (m, 1H), 3.40 (d, J = 6.8 Hz, 1H), 2.23 (dt, J = 13.3, 6.5 Hz, 1H), 2.11 (td, J = 14.2, 7.1 Hz, 1H). ^13^C NMR (101 MHz, DMSO-d_6_) 172.07, 158.82, 149.73, 148.48, 147.33, 138.90, 134.79, 130.71, 122.52, 121.05, 118.68, 117.58, 109.16, 107.95, 107.91, 101.64, 52.49, 50.37, 43.81, 29.62.

## **Cell lines and cell culture**

The K562 (RRID: CVCL_0004) and HEK-293T (RRID: CVCL_0063) (WT JAK2-expressing leukemia lines), and human JAK2-V617F-positive AML lines, HEL (RRID: CVCL_0001), SET2 (RRID: CVCL_2187) and UKE-1 (RRID: CVCL_0104), were obtained from the ATCC (Manassas, VA, USA) Cell Line Bank. K562-Luc^+^ (RRID: CVCL_0004) cells were obtained from Shanghai Model Organisms Center, Inc. HEL-Luc^+^ (RRID: CVCL_0001) cells were constructed in-house. Most of the cell lines were purchased between 2022 and 2023, while the HEL-Luc and K562-Luc cell lines were either purchased or constructed in 2024. All cell lines used in this study were cultured with 5% CO2 at 37°C, at a concentration of 2×10^5^ to 5×10^5^ in RPMI (Gibco, Thermofisher), with 10% FBS (VivaCell, C04002) and supplemented with 1% penicillin/streptomycin. SET-2 cells were cultured in RPMI with 20% FBS (VivaCell, C04002) and 1% penicillin/streptomycin. HEK-293T cells were cultured in DMEM with 10% FBS (VivaCell, C04001) and 1% penicillin/streptomycin. Human cell lines were authenticated within 6 months of manuscript preparation through cell line short tandem repeat (STR) profiling conducted by a certified Contract Research Organization (CRO), SHANGHAI BIOWING BIOTECHNOLOGY Co. LTD. All cell lines tested matched >85% with lines listed in the ATCC or DSMZ Cell Line Bank STR and were confirmed to be virus- and mycoplasma-free.

## **Biologic reagents**

The following antibodies were obtained from Cell Signaling Technology (Shanghai Universal Biotech Company): JAK2 (rabbit, #3230S), pSTAT3 (rabbit, #9131), STAT3 (rabbit, #4904), pSTAT5 (rabbit, #4322), STAT5 (mouse, #4807), AKT (rabbit, #9272) (mouse, #2920), HSP90 (rabbit, #4874), and anti-GAPDH (14C10) (rabbit mAb, #2118). All antibodies were used at 1:1000 for immunoblotting, with the exception of anti-GAPDH, which was used at 1:5000. DESI2 (rabbit, 20517-1-AP, 1:1000) and beta-Actin (mouse, 66009-1-Ig, 1:10000) antibodies were purchased from Proteintech group. FLAG-tag antibody (3B9) (M20008M, 1:1000), the secondary antibodies (Goat Anti-Rabbit lgG antibody, M21002L) and (Goat Anti-Mouse IgG HRP, M21001L) were obtained from Abmart. Ubiquitin (P4D1) (sc-8017), SUMO-1 (D-11) (sc-5308), SUMO-2/3/4 (C-3) (sc-393144), was purchased from Santa Cruz Biotechnology, Inc. and used at 1:1000 for immunoblotting. Normal rabbit IgG (#2729) for co-IP was obtained from CST. Protein A/G magnetic beads and Streptavidin beads were purchased from MedChemExpress (MCE, Shanghai). The Bright-Glo™ Assay Reagent was obtained from Promega (E2620). The cell viability detection kit (CellCounting-Lite 2.0 Luminescent Cell Viability Assay) was obtained from Vazyme (DD1101-02). The D-Luciferin, Potassium Salt was obtained from Yeasen Biotechnology. The immobilon western HRP substrate was purchased from Millipore (WBKLS0500). Different buffer solutions preparation was using the PH adjustment calculator from Mettler Toledo (FE28-Standard).

## **shRNA Knockdown and CRISPR Knockout assay**

The pLKO.1-puro lentiviral shRNA vector particles against DESI2 were obtained from Sigma-Aldrich (St. Louis, MO). Lentivirus package were carried out following PEI transfection manufacturer's instructions. Different leukemia cells were incubated with the viral particles in the presence of 5-8 μg/ml Polybrene (Hanbio) for 24 hours, and then fresh medium was added. 72h post-infection, the cells were selected with 0.3-1 μg/ml puromycin for 48 hours. Following selection, cells were used for the studies as described.

For CRISPR-CAS9 KO assay. DESI2 sgRNAs were designed and inserted into an all-in-one vector pLentiCRISPR V2 (addgene, #52961) following the manufacturer's instructions of the Dr. Feng Zheng’s Laboratory. HEL, UKE-1 and K562 cells were infected with Lentiviral sgRNAs and selected for puromycin resistance (0.3-1µg/ml) post-72h infection. Cells were collected after 3-5d of selection, and protein as well as mRNA levels were determined by immunoblotting and QPCR.

The sequences of the sh/sgRNAs are as follows:

| Name | Target sequences |
| --- | --- |
| sh15613 | CTGACAATTGCCAGATCTATG |
| sh67575 | GCTTTATCAGAGATTCTTTGT |
| sh68008 | CGGACTTCCTAGAAGATGATA |
| sh72609 | GAAAGAGATTCCTCGCTGGAT |
| sh68007 | CCACAGCAATAGAGCAAGTTA |
| DESI2-sgRNA1 (KO1) | GGAATTGAAGTCTATGGCAG |
| DESI2-sgRNA2 (KO2) | TTTTTCTTCAGCTTTATCAG |
| DESI2-sgRNA3 (KO3) | GCAAGTCGATTGATCCAGCG |

## **Immunofluorescence (IF) and confocal Microscopy**

DESI2 was stably knocked out in HEK-293T cells with sgRNA mixtures or SCR control, followed by transfected with an expression construct encoding EGFP fusion JAK2-V617F, post-24h transfection, cells were seeded and adhered overnight, then fixed in 4% formaldehyde for 0.5 h at room temperature (RT). Cells were then blocked with 1% BSA and permeabilized with 0.5% Triton X‐100, and incubated with antibodies to JAK2 and DESI2 for 1 hr. at RT. Washed cells with 1× PBS and incubated for 1h with anti-rabbit Alexa Fluor-594 Conjugate (CST, 8889S) in 1 × permeabilization buffer containing 10% normal human serum. Cell nuclei were stained by DAPl Stain Solution (Sangon Biotech, E607303, 1:5000). And slides were visualized and imaged with immunofluorescence microscopy or laser confocal microscopy.

## **Cell transfections**

HEK-293T cells were seeded and transfected at density ~70% using Polyethylenimine (PEI) (Polysciences) or Liposomal Gene Transfection Reagent (Meilunbio, MA0672) according to the manufacturer's instructions. The plasmids used were: FLAG-JAK2-V617F (provided by Prof. Wenchao Wang) or EGFP-JAK2-V617F (PPL, PPL01266-2d) and pLenti-CMV-DESI2-His-GFP-BSD (PPL, PPL03095-4a) were obtained from Public Protein/Plasmid Library.

## **Mass spectrometry-based proteomics**

We sought to investigate JAK2 complex through Co-IP followed with a mass spectrometry-based proteomic. Briefly, the method used to generate lysates for the JAK2 mass spectrometry study involved several steps: cell collection, lysis, protein extraction, and immunoprecipitation. Specifically, cells were harvested from culture dishes, washed twice with 1 × PBS to remove residual media, and then lysed in RIPA buffer containing protease and phosphatase inhibitors to prevent degradation and dephosphorylation. Protein concentration was measured using the BCA assay to ensure consistent input for subsequent analysis. Following protein quantification, the lysates were subjected to immunoprecipitation using an anti-JAK2 antibody to isolate JAK2-bound protein complexes. The immunoprecipitation was performed overnight at 4°C with gentle agitation, followed by washing steps to remove nonspecific binding. The purified JAK2 complexes were then eluted and processed for mass spectrometry analysis to identify interacting proteins and post-translational modifications. The quantitative mass spectrometry was performed and analyzed by Shanghai Omicsspace Biotech Co., Ltd.

For normalization, raw intensity values were processed using label-free quantification (LFQ) within MaxQuant. Additionally, peptide intensities were normalized to the total ion current (TIC) to correct for any variation across samples and allow for direct comparison between experimental groups. Proteins potentially interacting specifically with JAK2-V617F were ranked based on the number of unique peptides identified, with the top 50 proteins selected for further analysis. These include both newly identified proteins and previously reported JAK2-interacting proteins such as JOSD1 and HSP90AB1, which served as positive controls.

# **Supplementary Figures**

**Figure S1: DESI2 selectively binds mutant JAK2.** **(a)** A schematic overview of the research workflow. The study progressed through four key stages: (1) Target Discovery: Identification of DESI2 as a critical stabilizer of JAK2-V617F. (2) Mechanistic Validation: Defining DESI2‘s role as a deSUMOylase and deubiquitinase for JAK2-V617F at K962. (3) Therapeutic Intervention: Design and optimization of WWQ-03-012, a first-in-class small-molecule DESI2 inhibitor. (4) Preclinical Assessment: Demonstration that either genetic depletion of Desi2 or pharmacologic inhibition by WWQ-03-012 effectively promotes JAK2-V617F degradation and suppresses tumor growth across multiple MPN preclinical models. **(b)** Ba/F3-WT and Ba/F3-JAK2-V617F isogenic cells were subjected to Co-IP (IP–DESI2), followed by immunoblotting for JAK2, DESI2, and GAPDH. **(c)** K562 cells were transfected in parallel with FLAG–JAK2-WT or FLAG–JAK2-V617F (same pcDNA4.1 construct); Co-IP was performed with anti-FLAG antibody and analyzed for FLAG, DESI2, and GAPDH. Shown are the representative results of three independent experiments (n = 3).

**Figure S2: Knockdown of DESI2 selectively destabilizes mutant JAK2.** **(a)** DESI2 knockout (KO) HEK293T cells were generated as described in Figure 2 and transfected with or without the JAK2-V617F-EGFP fusion vector for 48 hours. Cells were then incubated on a microscope slide overnight. Representative fluorescence microscopy images (40× magnification) showed the JAK2-V617F-EGFP signal (left panel). Trans illumination was used to visualize the live cells. Scale bar: 20 μm. Objective quantification was performed to evaluate the JAK2-V617F-EGFP signal intensity (right panel). **(b)** mRNA levels of JAK2 and DESI2 were measured by qPCR for samples corresponding to Figure 2c. **(c)** DESI2 KD HEK293T cells were generated as described in Figure 2 and transfected with plasmids encoding wild-type (WT) JAK2 or JAK2-V617F, with or without DESI2. JAK2, DESI2, and GAPDH protein levels were analyzed by immunoblotting with the indicated antibodies. **(d)** HEK293T cells with DESI2 KD targeting the ORF sequence were generated as described in Figure 2. Monoclonal cells (#1, #3) were selected, immunoblotting shows protein levels of wild-type (WT) JAK2, DESI2, and GAPDH. **(e)** HEK293T cells with DESI2 KD targeting the 3’UTR sequence were generated. Monoclonal cells (#10, #11) were selected and transfected with plasmids encoding DESI2. Protein levels of WT JAK2, DESI2, and GAPDH were analyzed by immunoblotting. **(f)**  The DESI2 KD HEL cells were generated as described in Figure 2 and subsequently transfected with plasmids encoding either wild-type DESI2 or the catalytically inactive DESI2 C108S mutant. The protein levels of JAK2-V617F, DESI2, and GAPDH were assessed by immunoblotting. **(g)** DESI2 KD HEL (JAK2-V617F) cells were generated using shDESI2 (67575) or scrambled control (SCR), as described in Figure 2a, and treated with or without MG132. JAK2-V617F and GAPDH protein levels were analyzed by western blotting (left panel). Objective quantification was performed to evaluate JAK2-V617F levels (right panel). Shown are the representative results of three independent experiments (n = 3). Error bars represent the mean ± SD. Student’s t-test or Ordinary one-way ANOVA.

**Figure S3: DESI2 mediates deSUMOylation/deubiquitination of JAK2-V617F at K962 and K970.** **(a)** HEK293T cells infected with DESI2-silencing shRNAs or SCR lentivirus were transfected with JAK2-V617F plasmid. SUMOylated JAK2-V617F proteins were detected using the indicated antibodies following pull-down with JAK2 antibody. **(b)** Co-immunoprecipitation (Co-IP) was performed using IgG or JAK2 antibodies in HEL cells. DESI2 was detected in both the immunoprecipitated samples (IP) and cell lysates (Input) by western blotting with the indicated antibodies. **(c-d)** The samples from (**b**) were further analyzed by liquid chromatography-mass spectrometry (LC-MS). SUMOylation and ubiquitination sites on JAK2 were identified and predicted using the SUMOplot™ Analysis Program, with the modification sites shown as indicated. **(e)** DESI2 KD HEK293T cells, generated as described in Figure 2, were transfected with plasmids encoding either JAK2-V617F or the JAK2-V617F-K962A mutant for 24 and 72 hours. Ubiquitinated JAK2-V617F was detected via pull-down with a JAK2 antibody, followed by immunoblotting to analyze protein levels of JAK2, DESI2, and GAPDH using the indicated antibodies. **(f)** Co-IP was performed in HEK293T cells overexpressing DESI2-WT, DESI2-C108S, JAK2-V617F, or JAK2-V617F-K962A, using an anti-DESI2 antibody. JAK2 and DESI2 were detected in both the IP and input lysates by western blotting with the indicated antibodies. Shown are the representative results of three independent experiments (n ****= 3).

**Figure S4: AlphaFold3 modeling uncovered the mechanism driving the mutant selectivity of DESI2.** The binding modes of DESI2 with both wild-type (WT) JAK2 and JAK2-V617F were predicted using AlphaFold3. DESI2 interacts with the FERM domain of JAK2-WT (monomer) through the formation of hydrogen bonds, primarily involving Ser143_DESI2_-Lys253_JAK2_, Trp149_DESI2_-Tyr124_JAK2_, Glu148_DESI2_-Ser126_JAK2_, Gln156_DESI2_-Ser126_JAK2_. Similarly, DESI2 forms hydrogen bonds with JAK2-V617F (monomer), inducing conformational changes in JAK2-V617F. Notably, DESI2 interacts with amino acids from both the FERM and JH1 domains. In the FERM domain, key interactions include Asn18_DESI2_-Cys125_JAK2_, Tyr20_DESI2_-Leu145_JAK2_, Ser23_DESI2_-Asn249_JAK2_, Arg130_DESI2_-Gln242_JAK2_. In the JH1 domain, the interactions are concentrated on Ser160_DESI2_-Asn1111_JAK2_, Gln161_DESI2_-Gln1112_JAK2_, Gln164_DESI2_-Asn1109_JAK2_. Compared to JAK2-WT, the binding pattern of DESI2 to JAK2-V617F involves the formation of three additional hydrogen bonds, suggesting enhanced interaction and structural stabilization.

**Figure S5: Knockdown of DESI2 disrupts downstream signaling and inhibits cell growth by destabilizing mutant JAK2.** **(a)** The impact of DESI2 KD on JAK2-V617F protein levels and downstream signaling in JAK2-V617F-positive HEL cells was assessed by western blotting with the indicated antibodies. **(b)** The levels of JAK2 and downstream signaling proteins in DESI2 knockout (KO) JAK2-V617F^+^ UKE-1 cells were assessed by western blotting using the indicated antibodies. **(c)** DESI2-knockdown HEL cells were simultaneously transfected with FLAG-tagged JAK2-WT. Immunoblotting was performed for FLAG, JAK2, DESI2, and GAPDH. **(d)** Effect of DESI2 KD on JAK2-V617F protein levels and growth in HEL cells. The impact of DESI2 KD on the growth of JAK2-V617F-positive HEL cells was evaluated using a growth assay. **(e)** The effect of DESI2 knockdown (KD) on apoptosis in JAK2-V617F^+^ HEL cells was assessed using Annexin V staining and flow cytometry. Shown are the representative results of three independent experiments (n = 3). Data are presented as the mean ± SD. Ordinary one-way ANOVA.

**Figure S6: DESI2 deficiency inhibits JAK2 mutant tumor growth *in vivo*. (a-b)** HEL (JAK2-V617F) cells with stable DESI2 knockdown (shDESI2) or scrambled control (SCR), generated as described in Figure 2, were implanted subcutaneously in vivo (n = 7). Body weight and tumor volume changes were monitored over time, with tumor length and width measured every 2 days for 42 days post-injection using a digital caliper. **(c)** Kaplan-Meier survival analysis of xenograft mice (n = 7). **(d)** HEL-Luc-GFP cells were first established via transduction with a firefly luciferase-expressing lentivirus. Subsequently, DESI2 knockdown was achieved using shRNAs (72609, 67575), with SCR and Mock serving as controls. Luminescence signal was confirmed as indicated. Data are presented as the mean ± SD. Ordinary one-way ANOVA and log-rank test.

**Figure S7: DESI2 deficiency does not suppress JAK2 wild-type leukemia growth *in vivo*. (a)** K562-Luc-GFP (JAK2-WT) cells were first established via transduction with a firefly luciferase-expressing lentivirus and then with lentiviruses encoding DESI2-targeting sgRNAs (KO1, KO2) or a scrambled control (SCR). Luminescence was measured as indicated. **(b)** K562-Luc-GFP cells (SCR, KO1, KO2) were generated as (**a**), cells were then grown in a tail vein injection-based non-invasive *in vivo* bioluminescence model of leukemia (n = 6). Bioluminescent images of representative mice with matched initial leukemia burden are shown. **(c)** Total flux bioluminescence plotted as a graph. **(d)** Kaplan-Meier survival analysis of xenograft mice. **e.** Body weights of xenograft mice over 49 days. Data are presented as the mean ± SD or SEM.

**Figure S8: Chemical proteomics of SB1-F-70 identified DESI2 as a top “hit”. (a)** Chemical structure of SB1-F-70. **(b-c)** Competitive activity-based protein profiling combined with quantitative mass spectrometry identified DESI2 as a target of SB1-F-70 in HEK293T lysates. Data are presented as the mean ± SD. Ordinary one-way ANOVA.

**Figure S9: WWQ-03-012 exhibits stronger binding affinity and target effect for DESI2 than SB1-F-70. (a)** Dose-response analysis confirmed that SB1-F-70 inhibits DESI2 in a purified enzyme biochemical assay (Ub-AMC) with an IC50 of 31.52 μM. **(b)** RMSD analysis of binding mode of WWQ-03-012 and DESI2. **(c)** The DC50 of 03-012 for JAK2-V617F was determined using the JAK2-V617F-HiBiT assay in HEK293T cells. **(d)** Proteomic Analysis: Protein levels were assessed following DESI2 knockdown and WWQ-03-012 treatment in HEL (JAK2-V617F) cells, compared to untreated controls. After intersecting both datasets, 273 significantly altered proteins were identified. Proteins are ranked by SequenceNumber (descending), with gene names on the y-axis and the relative protein ratio on the x-axis. Bubble size represents peptide count, and color indicates -log10(p Value). JAK2 ranks 11th (Top 5%). **(e)** HEL (JAK2-V617F) cells were treated with 10 μM 03-012 for 24 hours or subjected to DESI2 knockdown. Protein levels of JAK family members, DESI2, and GAPDH were analyzed by western blotting using the indicated antibodies. Shown are the representative results of three independent experiments (n = 3). Error bars represent as the mean ± SD or SEM.

**Figure S10: WWQ-03-012 selectively targets JAK2-V617F-positive leukemia cells, and its combination with Ruxolitinib further enhances efficacy. (a)** Growth Inhibition 50s (GI50s) of 48-hour WWQ-03-012 treatment on both WT and JAK2-V617F-mutated MPN/AML cell lines (n = 4). **(b)** Treatment of JAK2-WT (K562) and JAK2-V617F-positive (HEL, DAMI) cells with WWQ-03-012 for 24h, the cell viability was determined by CellTiter-Glo® luminescent cell viability assay. **(c)** Immunoblotting: JAK2 and DESI2 protein levels following DESI2 inhibition by WWQ-03-012 or JAK2 inhibition by Ruxolitinib in mouse UT7-MPLW515L-driven MPN cells. **(d)** Proliferation studies: Effects of 48 or 72-hr WWQ-03-012 or Roxulitinib treatment on mouse UT7-MPLW515L-driven MPN cells. **(e)** HEL and Ba/F3-JAK2-V617F cells were treated with Ruxolitinib or WWQ-03-012 for 24 or 72 hours, and cell viability was assessed using the CTG assay as described in (**a**). **(f)** Proliferation studies: Assessment of the effects of 24-hour treatment with WWQ-03-012, Ruxolitinib, or their combination on Ba/F3-JAK2-V617F cells. **(g)** Western blots: JAK2 and downstream signaling levels in DESI2 inhibition by WWQ-03-012, or JAK2 inhibition by Ruxolitinib, or combination treatment (combo) in JAK2-V617F^+^ cells. **(h)** Western blots: JAK2 and downstream signaling levels in DESI2 KD HEL cells, with or without combination treatment with Ruxolitinib, in JAK2-V617F^+^ HEL cells. Shown are the representative results of three independent experiments (n = 3). Data are presented as the mean ± SD or SEM. Student’s t-test or ordinary one-way ANOVA.

**Figure S11: WWQ-03-012 exhibits efficacy against Ruxolitinib-resistant cells.** Proliferation studies: **(a)** Effects of 24-hour treatment with WWQ-03-012 or Ruxolitinib on cell lines under persistent conditions with JAK2-V617F mutations. RP: Ruxolitinib-persistent. **(b-c)** Proliferation studies: Effects of 24- or 72-hour treatment with WWQ-03-012 or Ruxolitinib on Ruxolitinib-resistant cell lines harboring JAK2-V617F mutations. RR: Ruxolitinib-resistant. **(d)** Proliferation studies: Effects of 24-hour treatment with WWQ-03-012 or Ruxolitinib on Ruxolitinib-resistant/persistent MPN patient (MPN-RR-4) with JAK2-V617F mutations. MPN-RR: Ruxolitinib-resistant MPN patient. Shown are the representative results of three independent experiments (n = 3). Error bars represent the ± SD or SEM. Ordinary one-way ANOVA.

**Figure S12: Combination of WWQ-03-012 and Ruxolitinib enhances therapeutic efficacy in JAK2-V617F-driven MPN. (a)** JAK2-V617F transgenic MPN mice at 8 weeks of age were treated with WWQ-03-012 (12.5 mg/kg, i.p., BID), Ruxolitinib (Ruxo, 50 mg/kg, i.p.) alone, or in combination with 03-012 (Combo). After 15 days of treatment, peripheral blood was collected, and representative peripheral blood smears (PBS) of the indicated groups of mice are shown. Typical red blood cells (RBCs), platelets (PLTs), and reticulocytes (RET) are indicated. Scale bar = 200 μm. **(b)** The percentage of Lin- Sca-1^+^ c-Kit^+^ (LSK) cells in bone marrow samples from vehicle, 03-012, Ruxolitinib, or combination-treated mice was measured by flow cytometry (n = 3-4). Error bars represent the ± SD or SEM. Ordinary one-way ANOVA.

**Figure S13: DESI2 inhibition does not affect JAK2-WT clones in littermate wild-type control mouse models. (a)** Validation of the effects of DESI2/JAK2 inhibition in littermate wild-type control mice. Statistical analysis of white blood cell (WBC), red blood cells (RBC), and platelet counts in peripheral blood from the indicated group of mice. Each dot represents one mouse (n = 6-8). **(b)** Flow cytometric analysis of TER119⁺ (erythroid) cell frequencies was performed to assess the impact of treatment on erythropoiesis in treated mice. **(c)** Flow cytometric analysis of B220⁺ (B lymphoid) and CD3e⁺ (T lymphoid) cell frequencies was performed to assess the impact of treatment on lymphopoiesis in treated mice. **(d)** Flow cytometric analysis of Gr1⁺Mac1⁺ (myeloid) cell frequencies was performed to assess the impact of treatment on myelopoiesis in treated mice. **(e)** The relative number of Lin- Sca-1^+^ c-Kit^+^ (LSK) cells in the bone marrow was assessed using flow cytometry and statistically analyzed following treatment with different conditions. Data are presented as the mean ± SD. Ordinary one-way ANOVA.
